# Recent Advances in Zirconium‐Based Metal–Organic Framework Membranes for Desalination

**DOI:** 10.1002/advs.75358

**Published:** 2026-04-17

**Authors:** Mao Fu, Hai‐Tian Xia, You Wu, Sen Li, Ying Liu, Ya‐Li Liu, Shi‐Qiang Wang, Yingchao Dong

**Affiliations:** ^1^ School of Geography and Environmental Science Guizhou Normal University Guiyang PR China; ^2^ Guizhou Provincial Key Laboratory for Prevention and Control of Emerging Contaminants Guiyang PR China; ^3^ College of Chemistry and Chemical Engineering Shanghai University of Engineering Science Shanghai China; ^4^ Research Center for Membrane and Film Technology Kobe University Kobe Japan; ^5^ Singapore Membrane Technology Centre Nanyang Environment and Water Research Institute Nanyang Technological University Singapore Singapore; ^6^ School of Science and Engineering The Chinese University of Hong Kong (Shenzhen), Longgang Shenzhen Guangdong China

**Keywords:** Desalination, membrane separation, MOF membranes, water treatment, Zr‐MOFs

## Abstract

Zirconium‐based metal–organic frameworks (Zr‐MOFs) have emerged as promising desalination membrane materials owing to their exceptional chemical and hydrothermal stability, tunable nanoporous architectures, and versatile surface functionality. This review summarizes recent advances in Zr‐MOF membranes for desalination, focusing on representative systems such as UiO‐66 and its derivatives, MOF‐801, MOF‐808, and PCN‐224. It highlights how framework chemistry, pore architecture, defect engineering, and membrane configuration collectively govern water transport and ion selectivity. Fabrication strategies are systematically discussed, covering both pure Zr‐MOF membranes and mixed‐matrix membranes (MMMs). Desalination performance is evaluated across various separation processes, including pressure‐driven filtration, forward osmosis, and pervaporation. Finally, we discuss the molecular‐level sieving mechanisms and outline future research directions toward the development next‐generation materials for sustainable water desalination.

## Graphical abstract

Zirconium‐based metal–organic framework membranes with tunable pore architectures, defect chemistry, and surface functionalities for Desalination are systematically reviewed. Their fabrication strategies, desalination performance across pressure‐driven, forward osmosis, and pervaporation processes, and structure–property–performance relationships are critically analyzed. This review provides a roadmap for designing next‐generation water purification membranes.

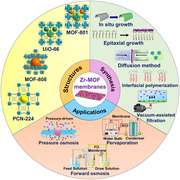

## 1 Introduction

Freshwater scarcity has become one of the most pressing global challenges of the 21st century, driven by population growth, industrialization, and climate change [1, 2, 3]. Desalination, a process that removes predominant ionic species (e.g., Na^+^, Mg^2^^+^, Ca^2+^, Cl^−^, HCO_3_^−^, SO_4_^2^^−^) and other impurities from seawater or brackish water to produce clean water, represents a promising strategy to mitigate global water scarcity [4, 5, 6]. Compared with conventional thermal processes, membrane‐based separation has proved to be a promising approach due to its merits such as low energy consumption, compact footprint, and high separation efficiency [7, 8]. Yet despite remarkable progress, state‐of‐the‐art polymeric membranes are fundamentally constrained by the long‐standing permeability‐selectivity trade‐off, limited resistance to extreme chemical environments, and vulnerability to fouling [9, 10, 11]. Continuous innovation in membrane materials is therefore crucial for improving desalination performance and meeting the rising demand for sustainable water purification [12]. Metal–organic frameworks (MOFs) have emerged as a compelling platform to meet these demands, owing to their crystalline porosity, molecular‐level tunability, and diverse chemical functionalities [13, 14, 15, 16]. Among them, zirconium‐based MOFs (Zr‐MOFs) stand out for desalination applications [17], as their sub‐nanometer pore apertures and high defect tolerance enable efficient ion sieving, fast water transport, and long‐term stability under harsh aqueous environments [18, 19, 20, 21, 22, 23]. Over the past two decades, Zr‐MOFs have demonstrated broad potential in adsorption and separation applications [24, 25, 26, 27, 28, 29, 30, 31, 32, 33, 34, 35, 36, 37, 38, 39, 40, 41, 42, 43, 44, 45, 46, 47]. In particular, Zr‐MOF‐based membranes have exhibited near‐ideal monovalent salt rejection, high water permeance, and stable operation under acidic, alkaline, or oxidative conditions [48, 49, 50, 51, 52, 53, 54]. Therefore, Zr‐MOFs provide a new opportunity to integrate precise pore geometry, programmable pore chemistry, and defect engineering into next‐generation membrane design.

Previous reviews have laid the foundation for understanding MOF‐based desalination membranes, either by including Zr‐MOFs as part of broader MOF families or focusing on specific materials such as UiO‐66 [48, 50, 55, 56, 57, 58, 59, 60, 61, 62, 63], yet none have provided a dedicated review on Zr‐MOF desalination membranes. To our knowledge, comprehensive analyses of representative Zr‐MOF platforms beyond UiO‐66, including MOF‐801, MOF‐808, and PCN‐224, and their progress in emerging desalination technologies remain lacking. Furthermore, a systematic comparison of how framework chemistry, defect engineering, and membrane configuration collectively govern water transport and ion selectivity has not been established. Given their unique advantages in defect engineering and structural stability, a focused review is clearly needed to fill this knowledge gap.

**FIGURE 1:**
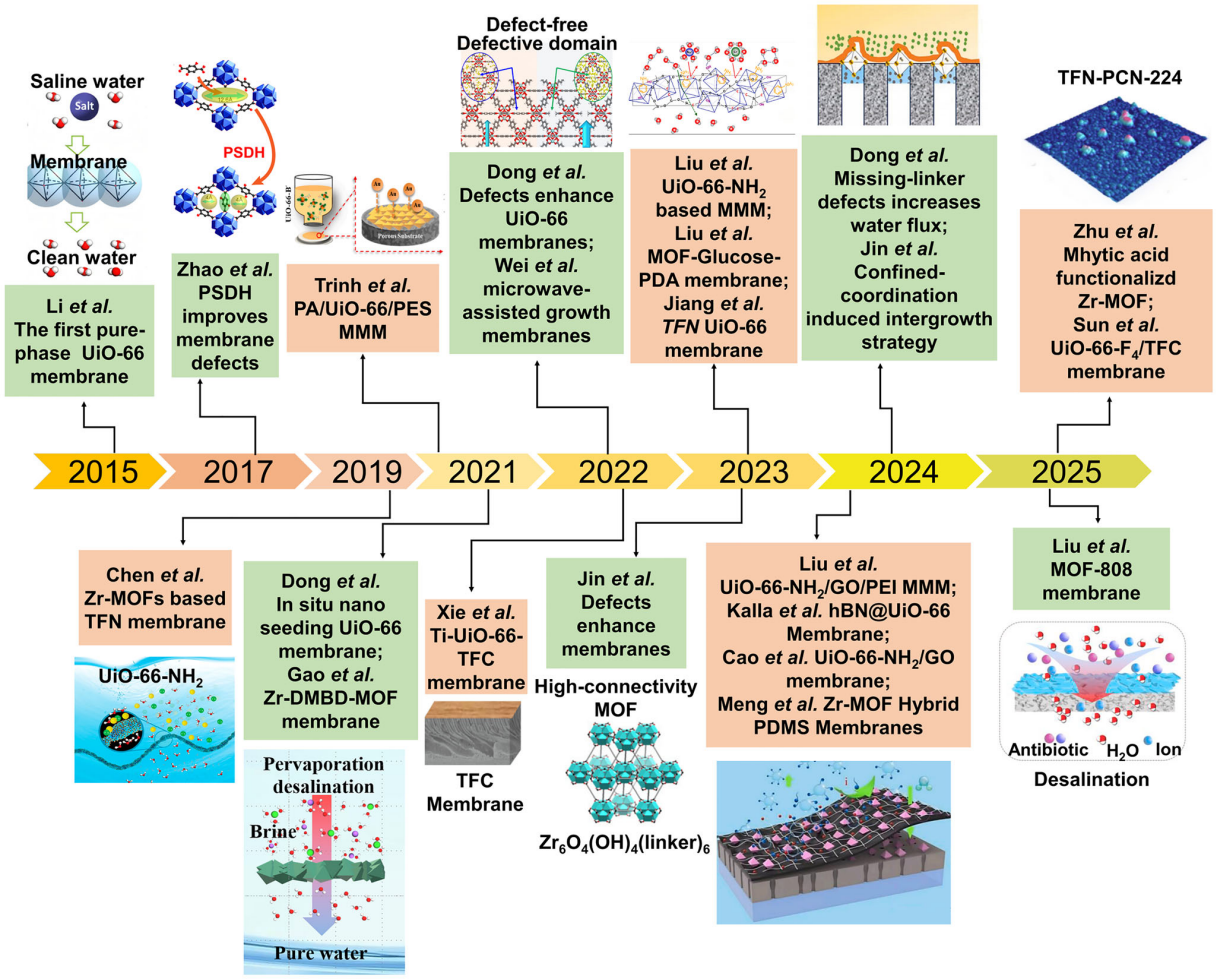
Development timeline of Zr‐MOF membranes for desalination [36, 64, 65, 66, 67, 68, 69, 70, 71, 72, 73, 74, 75, 76, 77, 78, 79, 80, 81, 82, 83, 84, 85, 86, 87, 88, 89, 90, 91, 92].

In this review, we highlight recent advances in Zr‐MOF membranes for desalination (Figure 1). We begin with a systematic overview of representative Zr‐MOFs, including UiO‐66 and its functional derivatives (e.g., UiO‐66‐NH_2_, UiO‐66‐SO_3_H), MOF‐801, MOF‐808, and PCN‐224, emphasizing their pore architectures, defect chemistry, and suitability for different desalination processes. We then categorize and evaluate fabrication strategies for Zr‐MOF desalination membranes, encompassing pure membrane construction (in situ solvothermal synthesis, epitaxial growth, diffusion methods, and emerging techniques) and mixed‐matrix membrane (MMM) design via interfacial polymerization, vacuum‐assisted filtration, and other approaches. Finally, we assess the performance of Zr‐MOF membranes across multiple desalination technologies, including pressure‐driven reverse osmosis (RO) and nanofiltration (NF), forward osmosis (FO), and pervaporation (PV). By offering a comprehensive and critical perspective, this review aims to provide new insights into the rational design of Zr‐MOF membranes and accelerate their development as next‐generation materials for addressing the global water crisis.

## 2 Representative Zr‐MOFs for Desalination Membranes

**FIGURE 2:**
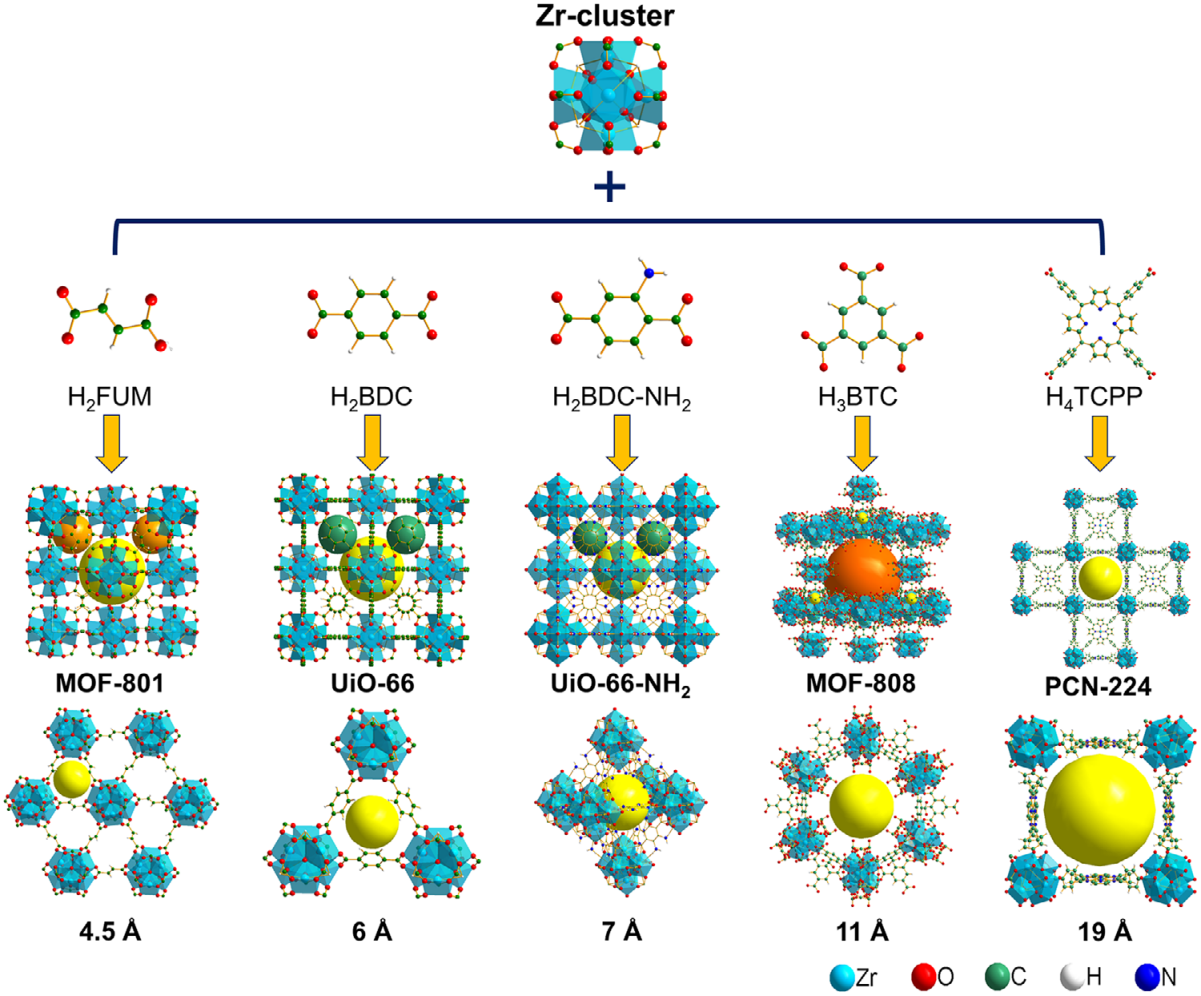
Crystal structures and pore aperture sizes of representative Zr‐MOFs desalination membranes.

To date, only a limited types of Zr‐MOFs has demonstrated the chemical robustness required for membrane‐based desalination. Representative examples include UiO‐66 and its functional derivatives [73, 84, 93, 94], MOF‐801 [79, 95, 96], MOF‐808 [83, 97, 98, 99], and PCN‐224 [70, 100], which collectively define the current material field for Zr‐MOF desalination membranes (Figure 2). Their exceptional stability originates from their Zr_6_O_4_(OH)_4_ secondary building units (SBUs), where highly charged Zr^4^^+^ ions form strong coordination bonds with carboxylate oxygen atom, in accordance with the hard–soft acid–base (HSAB) principle [63]. Beyond durability, these frameworks share structural attributes advantageous for desalination: sub‐nanometer pore apertures, rigid inorganic clusters, and hydrophilic internal surfaces enable precise water–ion discrimination, while defect tolerance allows controlled modulation of pore size and surface chemistry. Importantly, the ability of Zr‐MOFs to maintain crystallinity and pore connectivity under long‐term aqueous operation provides a prerequisite for constructing continuous, defect‐controlled membranes capable of sustaining high flux and high salt rejection. Despite the rapidly expanding library of Zr‐MOF structures, only a few have been successfully translated into desalination membranes. This limitation reflects the stringent and often competing requirements, including phase purity, scalable crystallization, mechanical integrity, and interfacial compatibility with supports or polymer matrices. In this context, UiO‐66‐type frameworks, MOF‐801, MOF‐808, and PCN‐224 have emerged as prototypical platforms, not only for their outstanding stability but also for their tunable pore architectures and amenability to defect engineering and surface functionalization. Below, we critically examine these representative Zr‐MOFs, focus on how their structures attributes dictate suitability for different desalination membrane. A comparative summary of their key structural parameters and stability profiles is provided in Table 1.

**TABLE 1:** Summary of the structural characters of Zr‐MOFs for desalination membranes.

Materials	Pore aperture (Å)	BET (m^2^·g^−^^1^)	Thermal stability (°C)	Chemical stability	Average particle size (nm)	Refs.
UiO‐66	6	1100 – 1600	500°C	pH 1–12, 500 h	∼200	[111]
UiO‐66‐NH_2_	7	876	300°C	Chlorine resistance (>1000 h)	∼60‐160	[112]
UiO‐66‐SO_3_H	5.8	235	400°C	Acid stability (pH 0–4)	∼200	[69]
MOF‐801	4.5	950	400°C	Acid and alkali (pH 1–12)	∼69	[113]
PCN‐224/Phytic acid	9.2	2297	400°C	Acid and alkali stability (pH 1–10)	∼220	[70]

UiO‐66 ([Zr_6_O_4_(OH)_4_(BDC)_6_]_n_) is the most extensively investigated Zr‐MOF for desalination membranes. Its exceptional chemical and thermal robustness derives from strong Zr─O coordination bonds [94], enabling stable operation in saline, acidic, and alkaline environments. From a separation perspective, UiO‐66's desalination capability is governed by its sub‐nanometer triangular pore windows (∼6 Å), which impose a steric barrier that excludes hydrated ions (e.g., Na^+^ ≈7.2 Å, Cl^−^ ≈6.6 Å) while permitting water transport. Beyond size selectivity, the framework exhibits a favorable hydrophilic–hydrophobic contrast that facilitates water uptake while mitigating membrane fouling [94]. Ligand functionalization offers a powerful strategy to tune transport properties. UiO‐66‐NH_2_, synthesized by substituting BDC with NH_2_‐BDC linkers, exhibits enlarged pore apertures (∼7 Å) due to ligand distortion and framework flexibility [101, 102]. The amino groups introduce hydrogen‐bonding sites that lower the activation energy for water diffusion, resulting in enhanced water permeance without compromising selectivity [103]. Conversely, UiO‐66‐SO_3_H achieves pore contraction (∼6.0 to ∼3.0 Å) through sulfonic acid functionalization, simultaneously enhancing hydrophilicity and negative surface charge. This modification improves size sieving capability and interfacial compatibility with polymer matrices, enabling efficient salt rejection while maintaining chemical stability [69]. Collectively, these examples demonstrate how pore geometry and surface chemistry can be tailored to balance water transport and ion selectivity, establishing UiO‐66 as a versatile platform for rational membrane design.

MOF‐801 ([Zr_6_O_4_(OH)_4_(FUM)_6_]_n_), constructed from shorter fumarate linkers, features a contracted pore architecture relative to UiO‐66. Its tetrahedral cavities (4.8 and 5.6 Å) and octahedral cavity (7.4 Å) are interconnected by pore windows of approximately 4.5 Å [104]. This reduced aperture lies well below the hydrated diameters of common monovalent ions, imposing a rigid steric barrier that enforces near‐complete ion exclusion. In contrast to UiO‐66, where rejection arises from a balance of size and charge effects [84], desalination in MOF‐801 is dominated by strict size sieving [105, 106]. Although partial dehydration would be required for ions to enter the sub‐5 Å windows, the high energetic cost associated with stripping hydration shells effectively suppresses ion transport. Consequently, MOF‐801 membranes achieve high salt rejection while maintaining water permeation through its interconnected nanocavities, making this framework particularly attractive for pressure‐driven demanding molecular‐level selectivity.

MOF‐808 ([Zr_6_O_4_(OH)_4_(BTC)_2_(HCOO)_6_]_n_) represents a hierarchical‐pore architecture with large adamantane‐like cages (∼18.4 Å) and smaller tetrahedral cavities (∼4.8 Å), interconnected by pore windows of ∼1.1 nm [107]. At first glance, such large apertures appear incompatible with efficient desalination, as they exceed the hydrated diameters of most mono‐ and divalent ions. However, MOF‐808 membranes utilize a combination of electrostatic exclusion, hydration‐layer formation, and defect‐mediated transport regulation to achieve selective desalination [108]. The abundance of carboxylate groups on the internal surface endows a strongly negative surface charge, generating a Donnan‐type electrostatic repulsion that preferentially suppresses multivalent anions such as SO_4_^2^^−^. Simultaneously, the highly hydrophilic pore environment promotes structured hydration layers that further hinder ion permeation while facilitating water transport. Importantly, defect engineering offers a route to enhance performance. Liu and co‐workers demonstrated that using trifluoroacetic acid (TFA) as a coordination modulator yields MOF‐808‐TFA membranes with controlled thickness and high density of hydrophilic defect sites, which introduce localized constrictions and additional water‐binding sites, effectively increasing ion transport resistance while preserving water permeability [83, 97]. This strategy highlights a critical design principle for large‐pore Zr‐MOFs, effective desalination relies not solely on pore size, but on the cooperative regulation of pore chemistry, defect density, and interfacial hydration.

PCN‐224 ([Zr_6_O_4_(OH)_4_(TCPP)_1.5_]_n_) represents a distinct class of functionally rich, large‐pore Zr‐MOFs. Constructed from 12‐connected Zr_6_ clusters and tetratopic porphyrinic ligands (TCPP), it forms an **ftw** topology framework with large mesoporous channels [109]. The high connectivity of the Zr_6_ nodes endows exceptional structural stability, while the porphyrinic moieties introduce intrinsic chemical functionality beyond conventional molecular sieving. The conjugated porphyrin units provide abundant polar and ion‐interactive sites, enabling adsorptive and electrostatic interactions that play a decisive role in ion separation [110]. Consequently, desalination in PCN‐224‐derived membranes is governed primarily by surface charge modulation and Donnan‐type exclusion rather than geometric confinement. To render PCN‐224 suitable for membrane‐based desalination, Zhu et al. [70] employed phytic acid functionalization to tailor its pore architecture and interfacial properties. This modification reduced the effective pore aperture from approximately 19 to ∼10 Å, increasing ion transport resistance while preserving high water permeability. Concomitantly, the introduced multivalent phosphate groups significantly enhanced the hydrophilicity and negative surface charge, as evidenced by a marked decrease in water contact angle from 77.5° to 64.9°. Although this modification led to a moderate reduction in specific surface area (from ∼2600 to ∼2297 m^2^·g^−^^1^), the enhanced polymer affinity facilitated the formation of dense, defect‐controlled mixed‐matrix membranes. The resulting modified PCN‐224 (mPCN‐224) membranes exhibited effective desalination performance dominated by electrostatic repulsion and charge‐selective ion exclusion.

## 3 Fabrication Strategies of Zr‐MOFs Desalination Membranes

The translation of Zr‐MOFs’ exceptional structural and chemical properties into high‐performance desalination membranes hinges critically on the fabrication strategy employed. Unlike conventional polymeric membranes, where separation performance is governed largely by chain packing and free volume, Zr‐MOF membranes derive their functionality from well‐defined crystalline nanopores. As such, the choice of fabrication method directly dictates key membrane attributes, pore continuity, defect density, crystallographic orientation, and interfacial compatibility, which collectively determine water permeance, ion selectivity, and long‐term operational stability. This section systematically examines the two principal routes for constructing Zr‐MOF desalination membranes: (i) pure crystalline membranes fabricated via in situ solvothermal growth, epitaxial growth, diffusion methods, and emerging techniques; and (ii) mixed‐matrix membranes (MMMs) formed by integrating Zr‐MOFs into polymer matrices through interfacial polymerization, vacuum‐assisted filtration, dip‐coating, and other approaches. For each strategy, we critically evaluate its principles, advantages, and limitations, with emphasis on how synthesis parameters translate into membrane microstructure and ultimately desalination performance. A comparative overview of these fabrication strategies is provided in Table 2.

**TABLE 2:** Summary of the preparation methods of Zr‐MOFs membranes for desalination.

Fabrication strategies	Advantages	Notes	Refs.
In situ Solvothermal Growth	Simple, strong adhesion, tunable defects	Large substrates; Scale: Industrial	[114]
Epitaxial Growth	Oriented, ultra‐thin, high selectivity	Gas separation; Complex; High energy	[115]
Diffusion method	Mild, fast, green	Fast membranes formation; Medium; Low solvent	[116]
Interfacial Polymerization	Ultra‐thin, defect‐free, low‐energy	Nanofiltration, pervaporation; Limited; Solvent recovery needed.	[117]
Vacuum‐Assisted Filtration	Simple, modular	Molecular sieves; Scalable; Low energy	[118]

### 3.1 Pure Zr‐MOF Membranes

**FIGURE 3:**
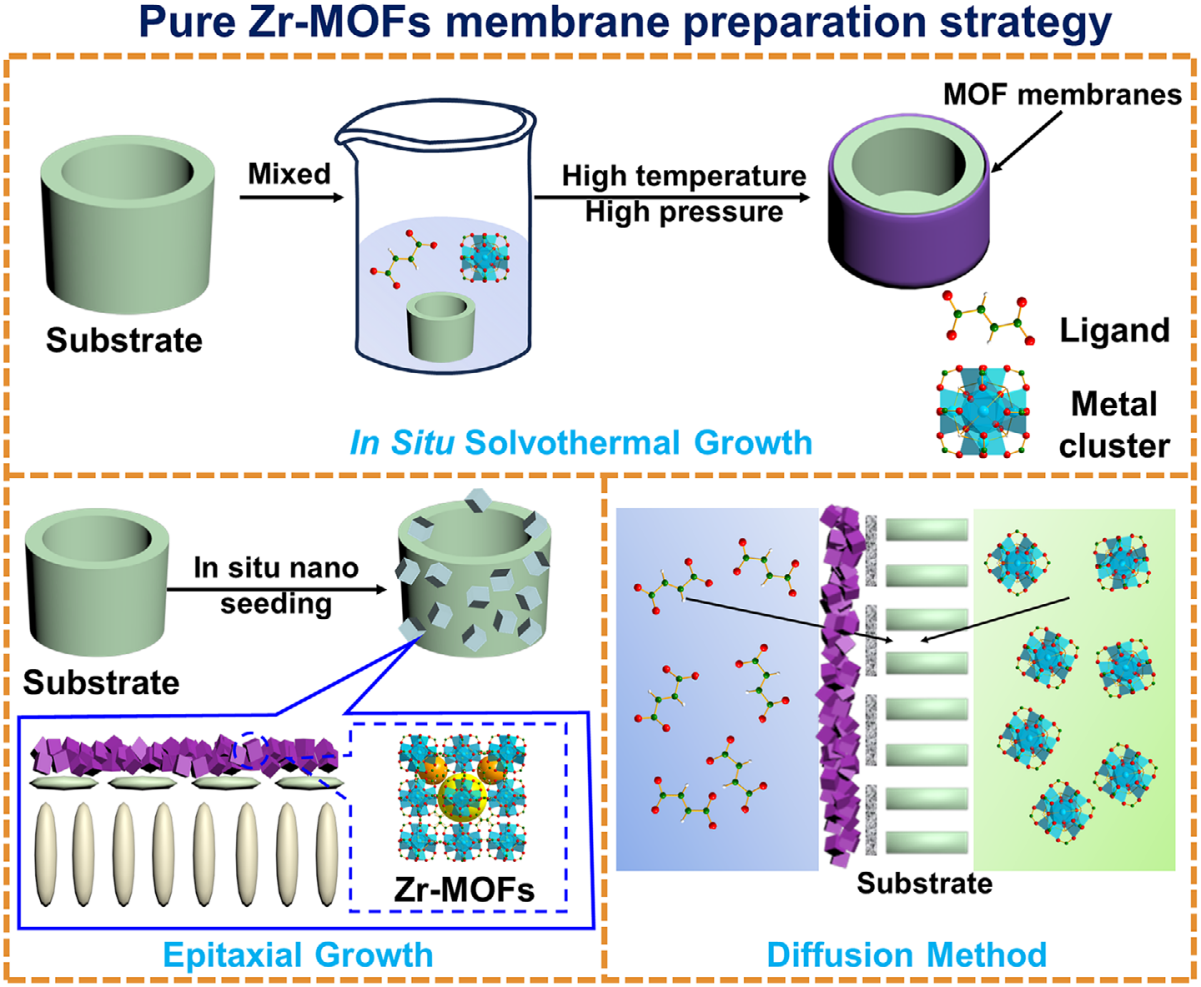
Schematic illustration of preparation strategies for pure Zr‐MOF membranes used in desalination, based on controlled crystal growth: in situ solvothermal growth, epitaxial growth, and diffusion method.

Pure Zr‐MOF membranes are typically constructed as continuous, intergrown, and polycrystalline layers anchored on porous substrates. Unlike polymeric membranes, where transport is governed by chain packing and free volume, the desalination functionality of Zr‐MOF membranes derives from well‐defined crystalline nanopores. Consequently, fabrication strategies directly determine pore continuity, defect density, and transport selectivity. Key structural parameters, cage dimensions, pore apertures, crystallographic orientation, and the exposure of Zr–oxo active sites, are highly sensitive to synthesis conditions. Variations in nucleation density, crystal growth kinetics, and framework–substrate interactions can alter membrane thickness, intercrystallite grain sizes, and defect distributions, thereby directly influencing water permeance, ion rejection, and long‐term stability. Thus, the fabrication strategy is not merely as a processing step but an essential tool for engineering transport pathways and regulating ion dehydration barriers. To date, several approaches has been developed to construct pure Zr‐MOF membranes, including in situ solvothermal growth, epitaxial growth, diffusion‐regulated assembly, as well as emerging techniques such as ultrasound‐ or microwave‐assisted synthesis and electrochemical deposition (Figure 3). Each method offers distinct advantages in controlling nucleation behavior, crystal intergrowth, and membrane compactness, while presenting unique challenges related to scalability, defect suppression, and substrate compatibility. Below, we critically evaluate these strategies, focusing on how growth mechanisms translate into membrane microstructure and desalination performance.

#### 3.1.1 In Situ Solvothermal Growth

**FIGURE 4:**
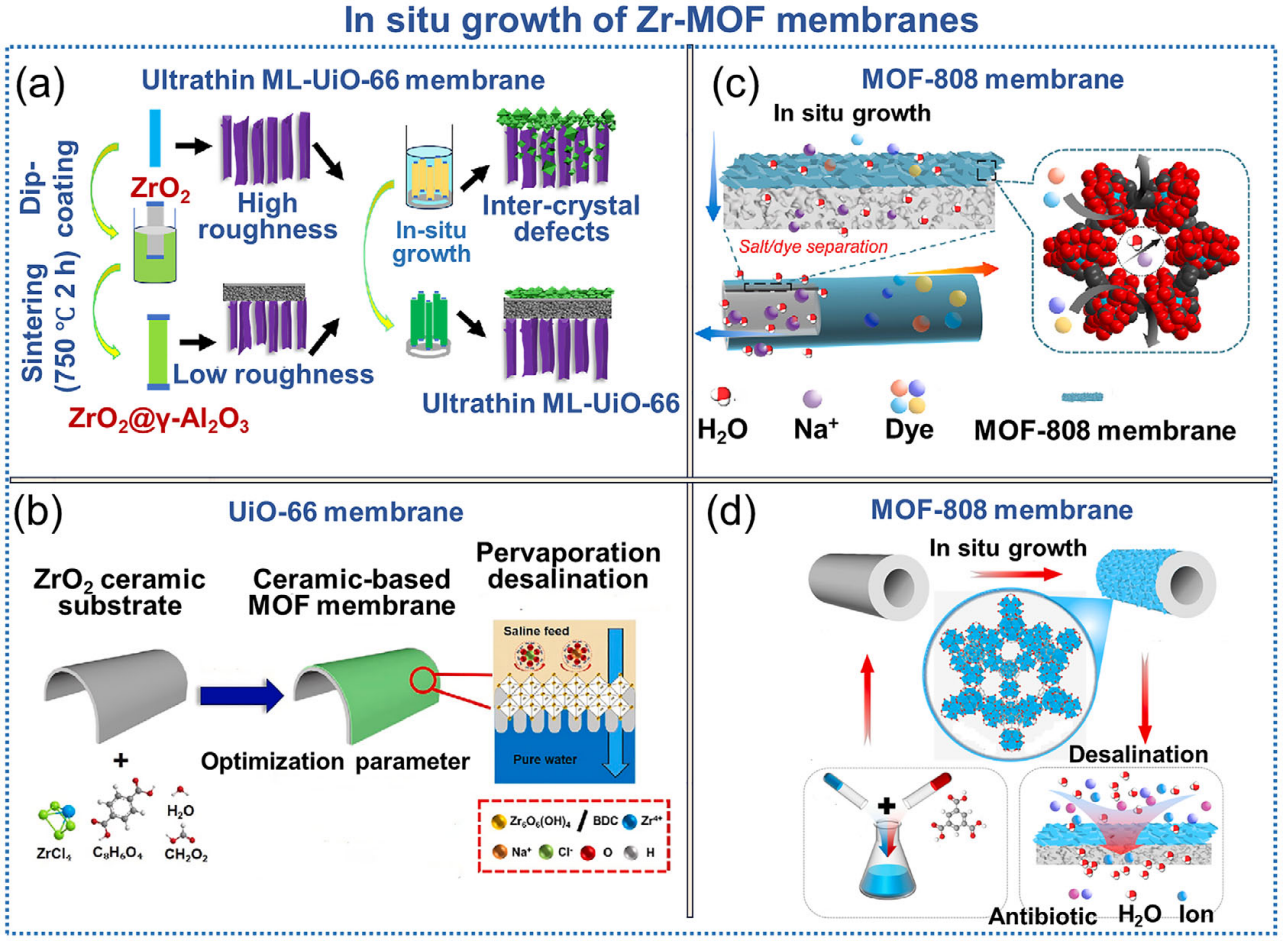
(a) Fabrication of γ‐Al_2_O_3_ interlayer and growth of ML‐UiO‐66 membrane [82]. (b) The process of structure optimization of ceramic‐based MOF membrane. Reproduced with permission [91]. Copyright 2024 Elsevier Ltd. (c) The desalination process of the MOF‐808 membrane. Reproduced with permission [83]. Copyright 2023 Elsevier. (d) Schematic illustration of ultrathin MOF‐808‐TFA membranes prepared by in situ modulation strategy for efficient antibiotic desalination [97]. Copyright 2025 Elsevier.

In situ solvothermal growth remains the most widely used method due to its simplicity and scalability [114]. Precursors are dissolved in a solvent system and undergo coupled nucleation and crystal growth directly on porous supports under elevated temperature and pressure [119]. The feasibility was first demonstrated by Li et al. (2015), who fabricated a continuous polycrystalline UiO‐66 membrane on alumina hollow fiber substrates, achieving a ∼2.0 µm thick defect‐free layer with grain sizes of 0.2–0.6 µm [84]. A persistent challenge is the trade‐off between membrane thickness and defect density. Dong et al. [82] introduced a γ‐Al_2_O_3_ interlayer to reduce surface roughness and increase nucleation sites, yielding ultrathin UiO‐66 membranes (∼103 nm) with missing‐linker (ML) defects that enhanced water transport while maintaining thermal stability (Figure 4a). The same group later optimized acetic acid as a modulator to tailor defect distributions, achieving ML‐UiO‐66 membranes with exceptional resistance to oxidative and alkaline environments in FO desalination (Figure 4b) [91]. However, heterogeneous nucleation on unmodified supports often leads to poor intergrowth and defects. A viable solution is support pre‐modification, for instance, Huang et al. used 3‐aminopropyltriethoxysilane as a covalent coupling agent to grow dense UiO‐66‐NH_2_ membranes on α‐Al_2_O_3_ tubes for PV desalination [87]. Beyond UiO‐66, this method has been extended to MOF‐808. Liu and co‐workers employed polyvinylpyrrolidone to increase nucleation density (Figure 4c) [83], and later used trifluoroacetic acid as a modulator to produce defect‐free MOF‐808 membranes (∼7.1 µm) with excellent hydrophilicity (contact angle 21.7°) for antibiotic desalination (Figure 4d) [97]. While versatile, in situ solvothermal growth suffers from poor control over nucleation kinetics and often requires multiple growth steps or support modification to achieve defect‐free membranes. The need for high temperatures, long reaction times, and large solvent volumes also raises scalability concerns. Nevertheless, its simplicity and directness make it a foundational method for laboratory‐scale demonstrations.

#### 3.1.2 Epitaxial Growth

**FIGURE 5:**
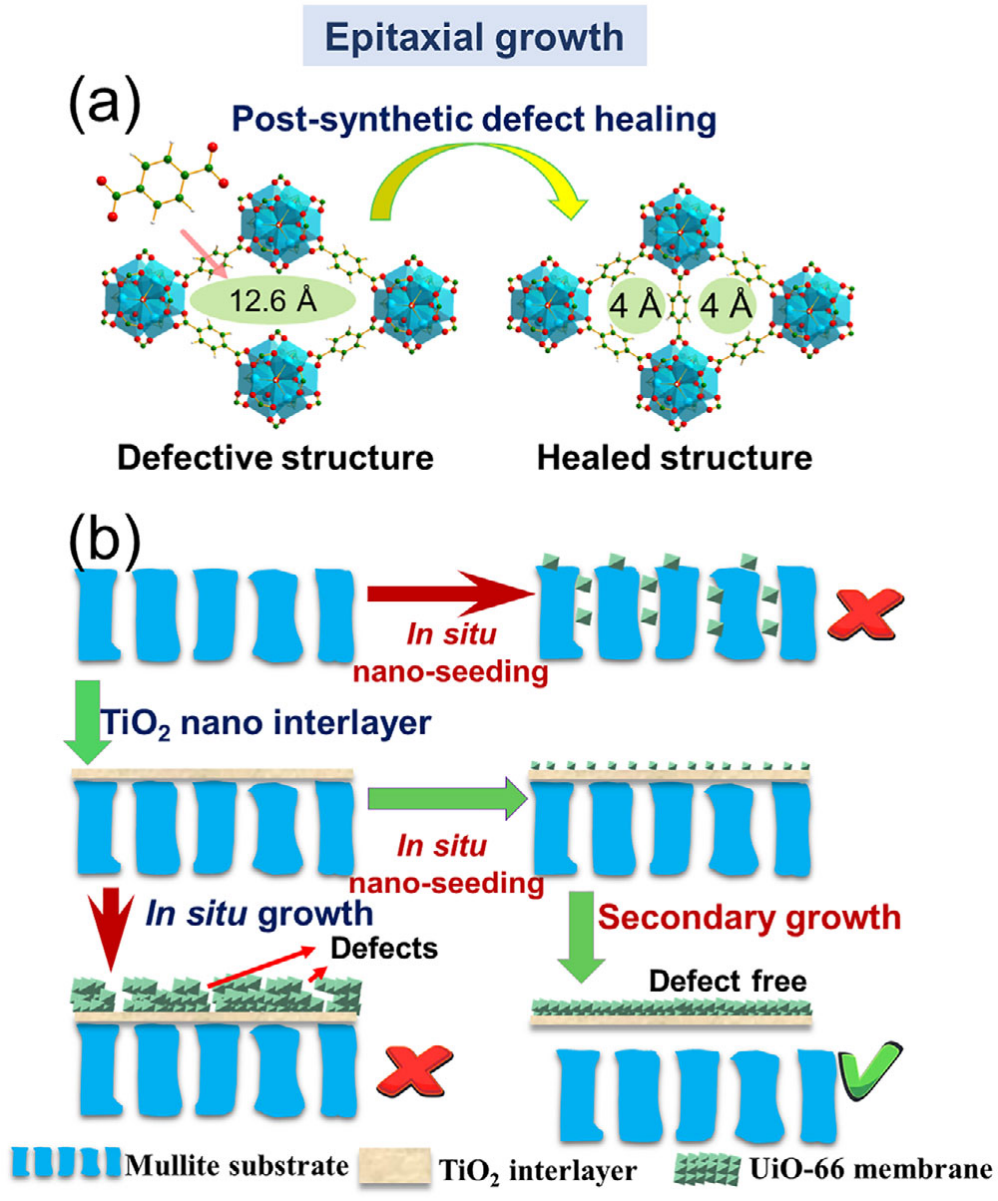
(a) Fabrication of UiO‐66(Zr)‐(OH)_2_ membrane via epitaxial growth and then post‐synthetic defect healing [90]. (b) Process for fabricating UiO‐66 membranes via a two‐step in situ seeding and secondary growth method [88].

Epitaxial growth decouples nucleation from crystal growth by first coating MOF seed crystals on the support, followed by secondary growth under milder conditions [120]. This strategy reduces nucleation energy barrier and facilitates continuous, defect‐free membranes [115]. In 2017, Zhao et al. [90] fabricated a UiO‐66(Zr)‐(OH)_2_ membrane of ∼3.5 µm thickness via epitaxial growth and applied post‐synthetic defect healing to improve salt rejection (Figure 5a). Dong et al. [113] developed a MOF‐801 membrane on ceramic hollow fibers using seed‐induced secondary growth with Span80 post‐treatment to prevent cracking during drying. Later, a two‐step strategy combining in situ nano‐seed deposition and epitaxial growth on TiO_2_‐modified mullite supports yielded a ∼500 nm thick UiO‐66 membrane with excellent PV desalination performance (Figure 5b) [88]. More recently, Jin et al. [121] employed an epitaxial growth method to deposit UiO‐66‐NH_2_, synthesized via a reverse diffusion method, onto a nylon substrate using vacuum filtration, followed by in situ growth to form a dense, defect‐free pure UiO‐66‐NH_2_ membrane. The introduction of –NH_2_ groups reduced the effective pore size from 5.6 Å (pristine UiO‐66) to 4.9 Å, thereby enhancing separation performance. Zeta potential measurements revealed a positive surface charge of 47.2 mV, approximately 1.5 times that of the unmodified UiO‐66 membrane, demonstrating that the UiO‐66‐NH_2_ membrane achieves superior desalination through synergistic size exclusion and Donnan effects. Epitaxial growth offers better control over membrane microstructure compared to direct solvothermal synthesis. However, it requires an additional seeding step, and the quality of the seed layer critically affects the final membrane. The method is also less amenable to continuous, large‐scale production due to its batchwise nature.

#### 3.1.3 Diffusion Method

**FIGURE 6:**
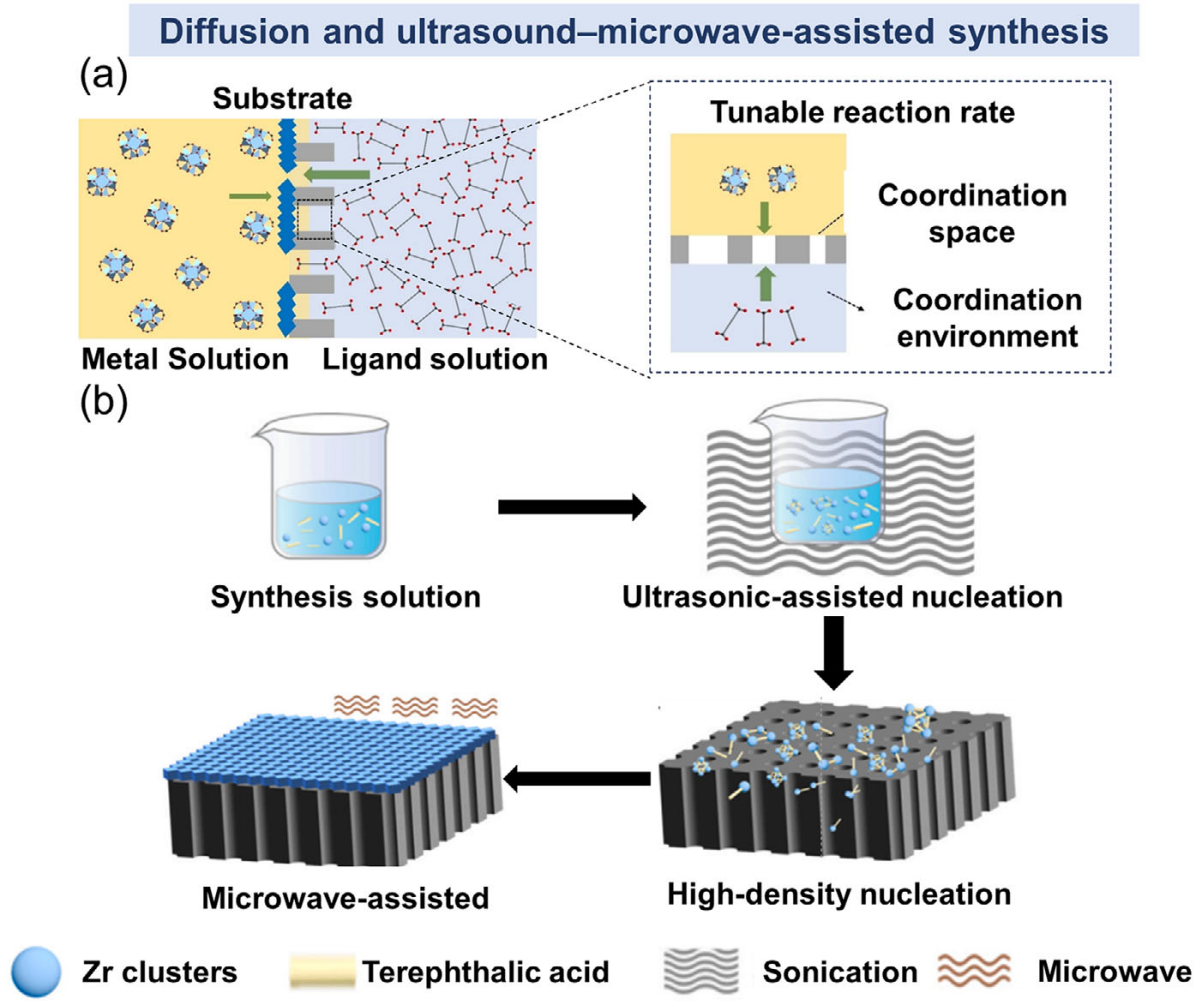
(a) Schematic illustration of (a) the confined diffusion synthesis strategy for growing continuous MOF membranes [79] (Copy Right 2024 Wiley) and (b) the ultrasonic‐/microwave‐assisted fabrication of UiO‐66 membranes [85] Copy Right 2022 Elsevier.

The interfacial diffusion method separates precursor solutions by a porous support, allowing the MOF layer to grow at the interface through the diffusion and subsequent coordination reactions. This approach is suitable for MOFs with fast reaction kinetics [116]. Jin et al. [86] employed a high‐probability coordination strategy to eliminate lattice defects in UiO‐66 membranes, achieving dense, ∼180 nm thick membranes with enhanced desalination performance. The strategy, achieved by precisely adjusting the stoichiometric ratio between linkers and metal clusters, successfully suppressed defect formation while improving separation performance. The same group later introduced a restricted‐coordination‐induced intergrowth strategy, producing ultrathin (∼100 nm), defect‐free MOF‐801 membrane with complete salt rejection and excellent stability under high temperature and pressure (Figure 6a) [79]. The diffusion method enables rapid and continuous membrane formation with precise thickness control. Its main limitation is the requirement for precise stoichiometric tuning and the difficulty in scaling to large‐area membranes due to concentration gradients. Additionally, the method is highly sensitive to support porosity and wettability, which can affect the uniformity of the resulting MOF layer. Nevertheless, its ability to produce ultrathin, defect‐free membranes under relatively mild conditions positions it as a promising route for fabricating high‐performance Zr‐MOF desalination membranes.

#### 3.1.4 Emerging Methods

Ultrasound–microwave‐assisted synthesis and electrochemical deposition offer rapid and energy‐efficient alternatives. Wei et al. [85] combined ultrasound to promote nucleation and microwave to accelerate growth, synthesizing an ultrathin UiO‐66 membrane (∼210 nm) in just 1 h for FO desalination (Figure 6b). Electrochemical deposition has also been explored for fabricating UiO‐66 membranes [81]. These methods reduce synthesis time and energy consumption, addressing a key limitation of conventional solvothermal routes. However, they remain at an early stage of development, with limited demonstration of defect control and long‐term stability. Further optimization is needed to achieve the same level of membrane quality as established methods.

### 3.2 Zr‐MOF‐Based Mixed‐Matrix Membranes (MMMs)

**FIGURE 7:**
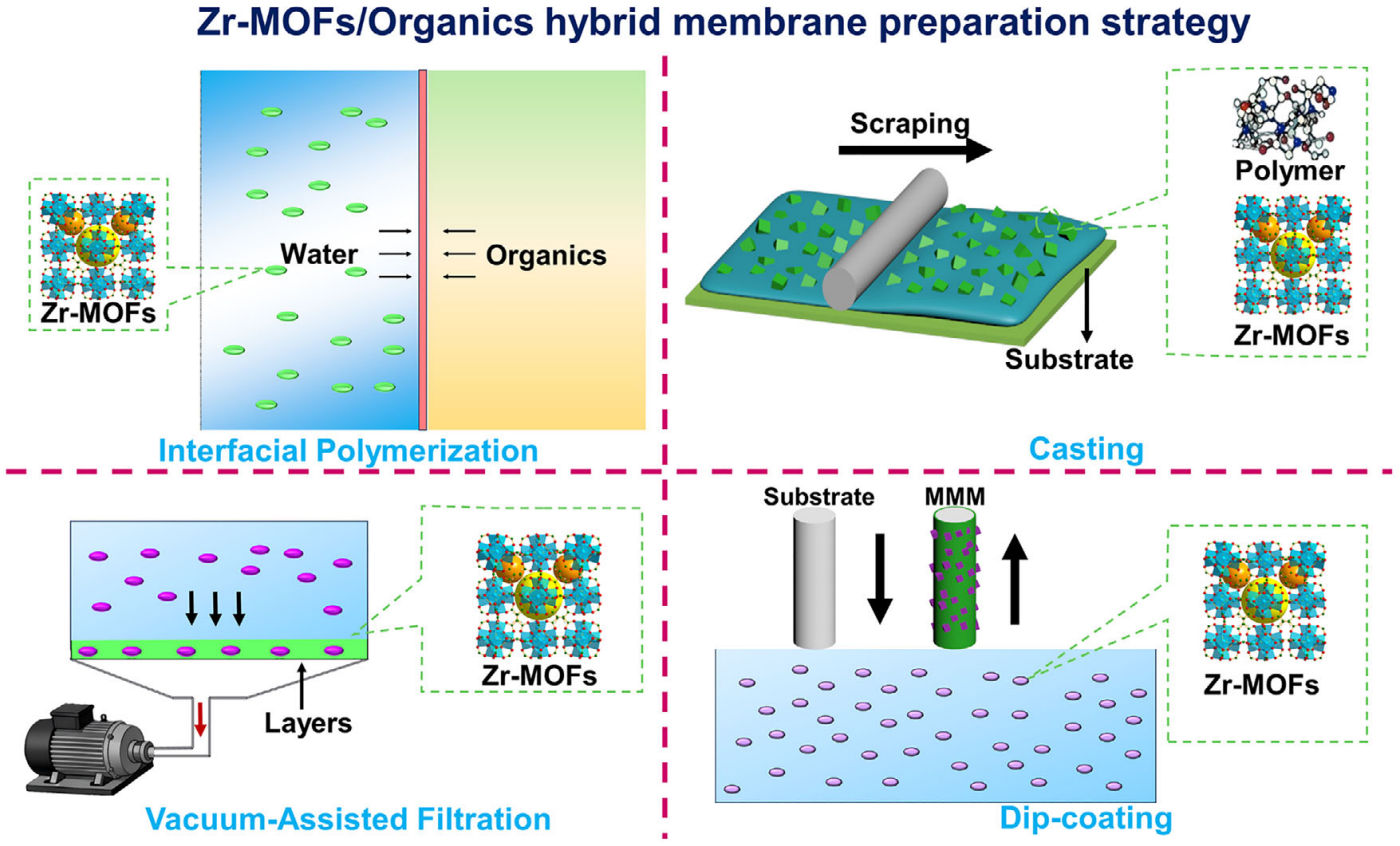
Preparation strategies of Zr‐MOFs/Organics hybrid membranes for desalination: Interfacial Polymerization, Casting, Vacuum‐Assisted Filtration, and Dip‐coating.

Beyond pure crystalline membranes, Zr‐MOFs have been extensively integrated into polymer matrices to form mixed‐matrix membranes (MMMs), offering an alternative and often more scalable route toward high‐performance desalination membranes (Figure 7). Unlike conventional inorganic fillers, Zr‐MOFs uniquely combine crystalline, tunable nanopores with organic‐inorganic hybrid chemistry, enabling simultaneous regulation of water transport pathways and membrane microstructure. Their structural diversity allows precise tailoring of pore size, surface functionality, and hydrophilicity, while their exceptional chemical stability ensures structural integrity under harsh conditions. More importantly, Zr‐MOFs expose abundant carboxylate groups, µ_3_‐O/µ_3_‐OH sites, and functionalized linkers, which can engage in coordination or hydrogen bonding with polymer chains. These interfacial interactions significantly enhance filler dispersion, suppress non‐selective interfacial voids, and improve mechanical integrity, critical factors that often limit the performance of traditional MMMs. From a transport perspective, incorporating Zr‐MOFs introduces well‐defined nanochannels that complement the intrinsic free‐volume pathways of polymers, enabling synergistic regulation of flux and selectivity. As a result, Zr‐MOF‐based MMMs have emerged as a versatile platform that integrates the molecular sieving and charge‐regulation capabilities of MOFs with the processability and scalability of polymers. Below, we systematically discuss representative fabrication strategies, interfacial polymerization, vacuum‐assisted filtration, casting, and dip‐coating, and critically analyze how filler distribution, interfacial chemistry, and composite microstructure govern desalination performance.

#### 3.2.1 Interfacial Polymerization (IP)

Interfacial polymerization is the most widely used method for fabricating thin‐film composite (TFC) membranes, typically forming a polyamide (PA) layer on a porous support via reaction between an amine monomer and trimesoyl chloride (TMC) [117]. However, conventional PA‐TFC membranes are constrained by the permeability–selectivity trade‐off and fouling susceptibility [122]. Incorporating hydrophilic nanomaterials can alleviate these issues, but poor compatibility with the polymer matrix often reduces selectivity [56]. Zr‐MOFs have emerged as effective nanofillers to address these limitations. Han et al. [123] incorporated UiO‐66 nanoparticles into the PA layer via IP, achieving tunable membrane thickness (∼230–1058 nm) and significantly enhanced hydrophilicity (contact angle reduced from 47.8° to 23.9°). The resulting TFN membranes exhibited improved water flux and reduced reverse solute diffusion. Zhu et al. [124] developed a fish‐net‐like wrinkled PA morphology by loading UiO‐66‐NH_2_ onto a support via vacuum filtration prior to IP. The MOF aggregates served as robust nodes connecting continuous PA stripes, creating a rough, high‐surface‐area structure that increased effective filtration area and hydrophilicity. Covalent bonding between the MOF's amino groups and TMC further enhanced filler–matrix compatibility. Similarly, Liu et al. [125] modified UiO‐66‐NH_2_ with stearoyl chloride to improve dispersion in the organic phase, yielding a uniform PA layer (∼380 nm) with enhanced mass transfer channels.

**FIGURE 8:**
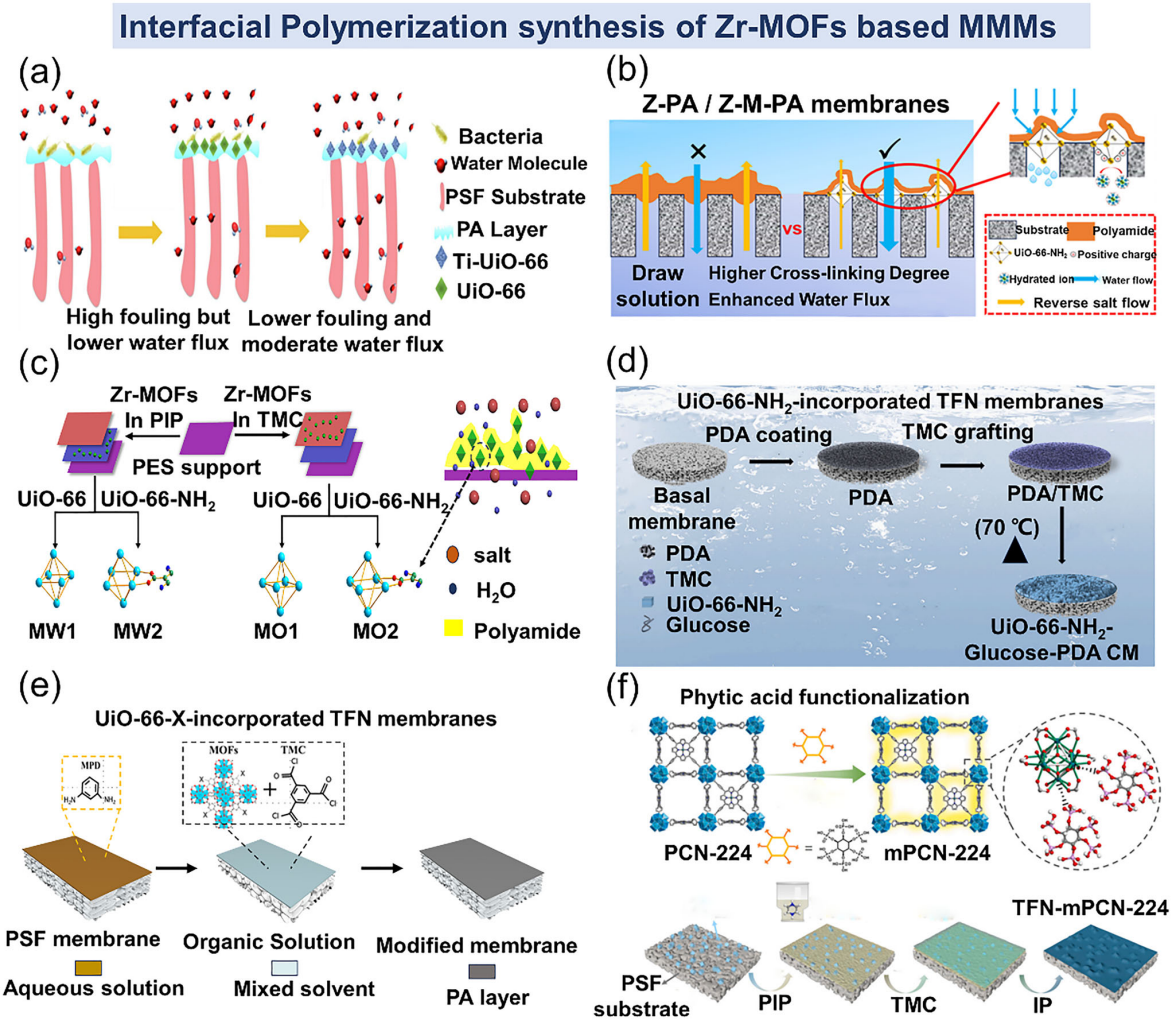
Synthesis of Zr‐MOF MMMs via interfacial polymerization. (a) Surface hydrophilicity modification of thin‐film composite membranes with Ti‐UiO‐66. Reproduced with permission [68]. Copyright 2022 Elsevier. (b) The mechanism for improving the water treatment performance of FO membranes [36]. Copy Right 2024 Elsevier Ltd. (c) The process of incorporating porous Zr‐MOFs into TFN membranes to enhance the desalination performance of the membranes; M: mass fraction, W: the addition of nano particles was in the aqueous phase, O: the nanoparticles were added in the organic phase [73]. (d) Schematic of the stepwise synthesis of the high‐performing biopolymer‐based membrane with a 3D‐integrated Zr‐MOF‐Glucose polydopamine (PDA) separation layer (CM in the diagram refers to composite membrane). Reproduced with permission [75]. Copyright 2023 Elsevier. (e) The synthetic procedure of the UiO‐66‐X‐incorporated TFN membranes [69]. Copy Right 2023 Wiley. (f) Synthesis route of phytic acid modified PCN‐224 nanoparticles and schematic diagram of TFN‐mPCN‐224 membrane fabrication. Reproduced with permission [70]. Copyright 2025 Elsevier.

Beyond UiO‐66, other Zr‐MOFs have been explored. Bonnett et al. incorporated PCN‐222 nanorods modified with myristic acid (MA) into the aqueous phase prior to IP [126]. The MA‐functionalized pores created rapid water transport pathways while maintaining high NaCl rejection (96.0%) at low loading (0.01 wt.%). Xie et al. [68] introduced Ti‐modified UiO‐66 into the TMC phase, achieving membranes with high NaCl rejection, enhanced water flux, and improved fouling resistance (Figure 8a). Dong et al. [36] deposited a UiO‐66‐NH_2_ nano‐seed layer on a ceramic substrate before IP, yielding thinner PA layers with higher crosslinking density and enhanced stability under extreme conditions (Figure 8b). Chen et al. [73] systematically compared the introduction of UiO‐66 and UiO‐66‐NH_2_ into either aqueous or organic phases during IP (Figure 8c). Subsequently, Liu et al. [75] proposed a sustainable strategy for fabricating biopolymer‐based membranes via cooperative IP using natural glucose and Zr‐MOFs‐UiO‐66‐NH_2_ (Figure 8d). To overcome the intrinsic flux–selectivity trade‐off of conventional polyamide membranes, He et al. [69] developed sulfonated UiO‐66‐SO_3_H–polyamide (PA) thin‐film nanocomposite (TFN) membranes, in which the incorporation of functionalized Zr‐MOF nanoparticles fundamentally reshaped both membrane morphology and interfacial chemistry. Relative to pristine TFC membranes, the UiO‐66‐SO_3_H‐based TFN membranes simultaneously retained high salt rejection while delivering markedly enhanced water flux (Figure 8e). The performance enhancement can be attributed to the dual role of UiO‐66‐SO_3_H nanoparticles within the PA separation layer. Compared with earlier UiO‐66‐based TFN membranes reported by Aghili et al. [127], the sulfonated UiO‐66‐SO_3_H‐PA TFN membranes exhibited a pronounced increase in water flux, while maintaining salt rejection rates exceeding 90%. Furthermore, Zhu et al. [70] functionalized PCN‐224 with natural organic phosphoric acid phytate, which reduced the pore size and enhanced its affinity for PA. They then employed electrophoresis deposition (EPD) combined with vacuum filtration‐assisted interfacial polymerization (VF‐IP) to synthesize nanocomposite membranes containing mPCN‐224 on polysulfone (PSF) substrates (Figure 8f). The introduction of modified PCN‐224 significantly reduced the membrane thickness and crosslinking density, decreasing the membrane thickness from 123 nm to 61 nm, while notably improving the salt‐selective flux.

IP offers unparalleled advantages in fabricating ultrathin, defect‐free selective layers with high scalability. However, achieving uniform dispersion of Zr‐MOF nanoparticles remains challenging, and excessive loading can induce agglomeration or interfacial defects. The compatibility between MOF surface chemistry and the polymer matrix is critical; functionalization strategies (e.g., amino, sulfonic, or fluorinated groups) have proven effective but add complexity. Future efforts should focus on optimizing MOF loading, dispersion, and interfacial bonding to maximize performance while maintaining mechanical integrity.

#### 3.2.2 Vacuum‐Assisted Filtration

Vacuum‐assisted filtration has gained attention as a simple and efficient technique for fabricating MMMs, particularly for layered or Janus structures [118]. Trinh et al. [67] first deposited UiO‐66 onto a Polyethersulfone (PES) support via vacuum‐assisted filtration, followed by IP to form a PA layer, achieving improved water flux due to the hydrophilicity of UiO‐66 [128]. Cao et al. [76] embedded UiO‐66‐NH_2_ and silver nanowires into a graphene oxide (GO) matrix to create a Janus membrane for membrane distillation (MD). The Janus structure enabled efficient removal of accumulated surfactants under intermittent voltage, maintaining stable performance over 170 h. Jin et al. [129] deposited a MOF‐801@GO composite on a polyvinylidene fluoride (PVDF) substrate via vacuum filtration, forming ultrathin MMMs (∼350 nm) with a water contact angle of 38.6°, achieving 99.99% salt rejection in direct contact MD. Vacuum‐assisted filtration enables rapid, scalable fabrication with precise control over membrane thickness and structure, particularly for 2D material‐based laminates. However, the method is highly dependent on substrate porosity and wettability, and achieving uniform dispersion without agglomeration remains challenging. The mechanical stability of the deposited layer under operating conditions also requires careful optimization.

#### 3.2.3 Other Methods

**FIGURE 9:**
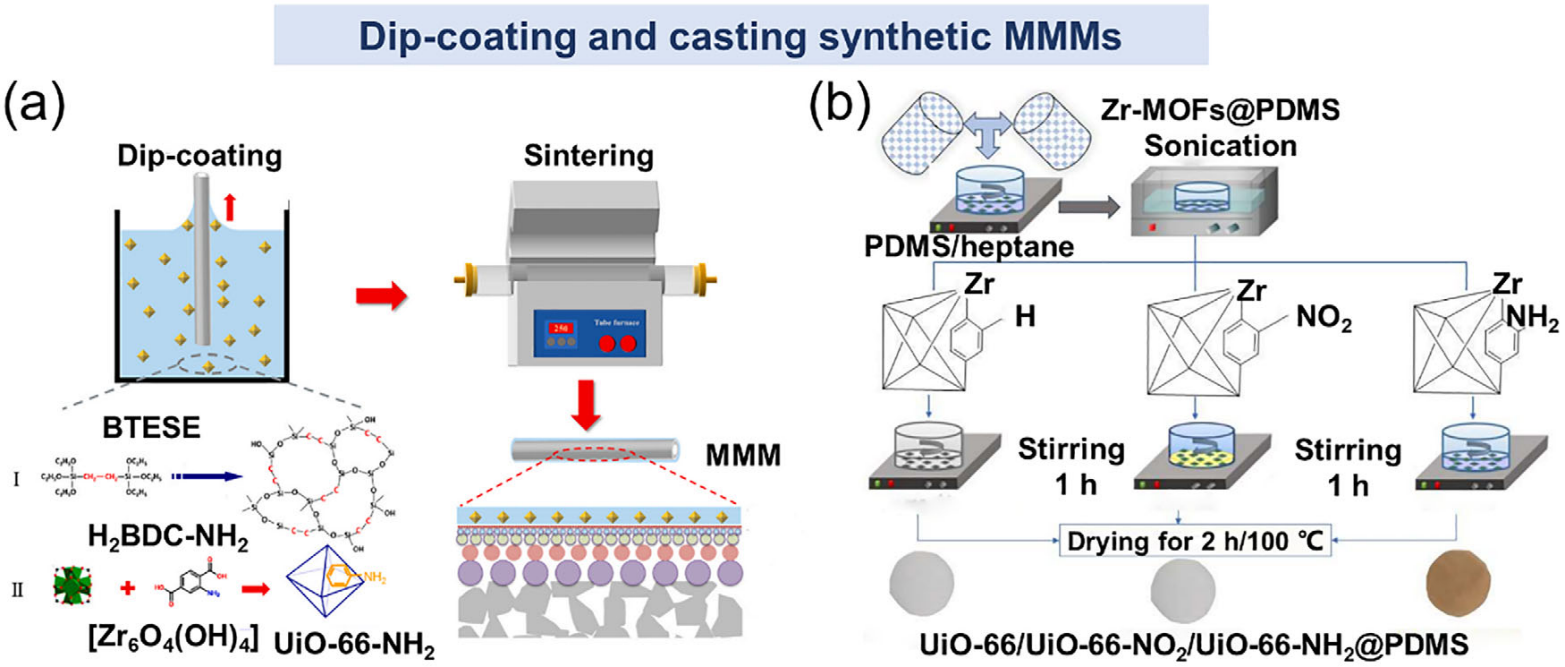
Synthesis of Zr‐MOF MMMs using dip‐coating and casting. (a) Schematic of fabrication of UiO‐66‐NH_2_/organosilicon MMM. Reproduced with permission [130]. Copyright 2023 Elsevier. (b) Schematic procedure for the synthesis of Zr‐MOF/PDMS membranes. Reproduced with permission [74]. Copyright 2024 MDPI.

Dip‐coating, casting, and phase inversion have also been explored for fabricating Zr‐MOF‐based MMMs. In 2023, Liu et al. [130] employed a dip‐coating method to deposit UiO‐66‐NH_2_/dual‐1,2‐(triethoxysilyl)ethane (BTESE) sol on an α‐Al_2_O_3_ support, forming membranes (∼200 nm) with excellent long‐term stability in PV desalination (Figure 9a). Li et al. [74] utilized casting to prepare a UiO‐66‐NH_2_ hybrid polysiloxane (PDMS) separation membrane with uniform MOF dispersion, achieving efficient separation of Na^+^/Ca^2^^+^ ions (Figure 9b). Additionally, Kalla et al. [65] employed the phase inversion method to incorporate UiO‐66 modified on hexagonal boron nitride (hBN) surfaces into a PVDF modified hollow fiber (HF) membrane. The resulting h‐BN/UiO‐66/PVDF MMM, with a thickness of around 30 µm, exhibited a finger‐like sponge‐like structure and maintained its integrity. The membrane also demonstrated excellent desalination performance during MD process. Dip‐coating and casting offer simplicity and versatility but often result in thicker membranes with limited thickness control. Phase inversion enables diverse membrane morphologies but is susceptible to defect formation. These methods are generally less scalable than IP for high‐performance desalination applications but remain valuable for specialized configurations, such as hollow fibers or thick‐film separators.

## 4 Desalination Processes and Structure–Performance Relationships

Membrane desalination technology utilizes selective separation membranes to efficiently remove dissolved salts and impurities under an applied driving force, serving as the core separation process for producing freshwater or low‐salinity water. This technology offers advantages such as low energy consumption, modularity, and environmental friendliness, making it a crucial solution for alleviating global water scarcity. In recent years, Zr‐MOF‐based separation membranes have demonstrated breakthrough performance in seawater desalination and industrial wastewater treatment through structural optimization [63]. To provide a coherent and mechanism‐oriented evaluation, this section systematically summarizes the desalination performance of Zr‐MOF‐based membranes according to their dominant driving forces, rather than individual material systems. Specifically, we examine (i) pressure‐driven processes, including reverse osmosis (RO) and nanofiltration (NF), where separation is governed by steric exclusion and dehydration energetics; (ii) osmosis‐driven processes, such as FO, where internal concentration polarization and water–solute selectivity play decisive roles; and (iii) thermally driven pervaporation (PV) processes, in which vapor‐phase transport through sub‐nanometer channels enables near‐complete salt rejection. This classification framework facilitates a direct comparison of structure–performance relationships and highlights how distinct Zr‐MOF architectures and membrane configurations are optimally matched to specific desalination modalities.

### 4.1 Pressure‐Driven Desalination

**FIGURE 10:**
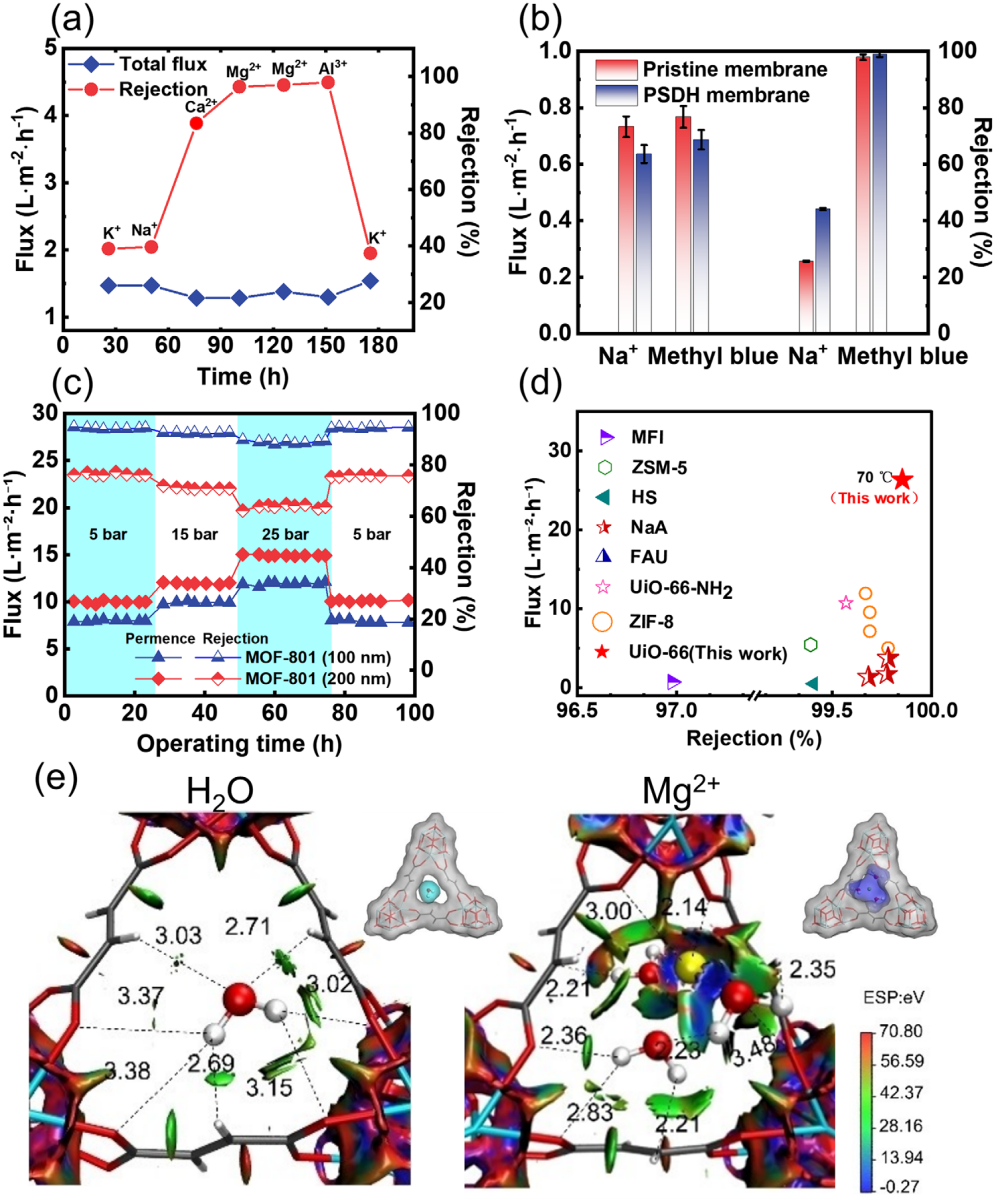
Desalination performance of Zr‐MOF membranes in pressure‐driven process. (a) The desalination performance of UiO‐66 membranes was tested at room temperature with a pressure difference of 10 bar using five different salt solutions of the same concentration [84]. (b) Separation performance of the UiO‐66 membrane before and after PSDH [90]. (c) Water flux and MgCl_2_ rejection of MOF‐801 membranes (feed concentration: 0.1 wt.%) [79]. (d) Water desalination performance comparison with past literatures [79]. (e) The 1*sign(λ2) mapped RDG (reduced density gradient) isosurface of the MOF‐801‐H_2_/CO_2_ and H_2_O and Mg^2+^ systems with isovalue of 0.5 and the superimposed Bader defined van der Waals (vdW) surfaces of MOF‐801 and molecules [79].

Pressure‐driven membrane processes use the pressure difference between the applied external pressure and the solution's osmotic pressure as the driving force for selective separation via permeable membranes [131]. In 2015, Li et al. [84] first synthesized a pure UiO‐66 membrane on a ceramic porous support using in situ solvothermal growth. This membrane showed limited rejection of monovalent ions, primarily due to ligand dynamics and potential linker defects. However, it exhibited high rejection for divalent metal ions through size‐sieving mechanisms, with rejection rates of 86.3% for Ca^2^^+^, 98.0% for Mg^2^^+^, and 99.3% for Al^3^^+^ (Figure 10a). Benefiting from the excellent chemical stability of UiO‐66, the membrane maintained stable separation performance during a 170 h test with various salt solutions. Early studies revealed that pure Zr‐MOF membranes are prone to intrinsic crystal defects during synthesis, which can compromise separation performance. To address this issue, Zhao et al. [90] introduced a post‐synthetic defect healing (PSDH) strategy. Following the repair process, the resulting UiO‐66 membrane achieved a NaCl rejection rate of 45%, which marked a ∼75% enhancement over the pre‐repair value, while maintaining a stable water flux of 1.06 kg·m^−^^2^·h^−^^1^·bar^−^^1^. Additionally, the membrane demonstrated excellent separation performance for methyl blue and other dyes, with a rejection rate of 99.8% and a water flux of 0.23 kg·m^−^^2^·h^−^^1^·bar^−^^1^ (Figure 10b).

To mitigate the intrinsic trade‐off between water flux and salt rejection, He et al. [69] engineered a thin‐film nanocomposite (TFN) membrane by incorporating‐SO_3_H‐functionalized UiO‐66 (UiO‐66‐SO_3_H) into the polyamide (PA) selective layer. The introduction of UiO‐66‐SO_3_H fundamentally altered the transport landscape of the PA layer, giving rise to additional hydrophilic nanochannels that facilitated rapid water permeation. As a result, the TFN membrane achieved a water flux of 3.47 L·m^−^^2^·h^−^^1^, corresponding to an enhancement of approximately 247% relative to the pristine TFC membrane. Beyond flux enhancement, the UiO‐66‐SO_3_H fillers played a critical role in preserving high separation selectivity. The uniform sub‐nanometer pore apertures of UiO‐66‐SO_3_H (∼3 Å), together with the strongly hydrophilic and negatively charged –SO_3_H groups, imposed an effective barrier against hydrated ions through a combination of size sieving and electrostatic exclusion. Consequently, the TFN membrane maintained a high salt rejection rate of 94.7% while simultaneously delivering substantially increased water flux. Jin et al. [79] synthesized a highly interconnected, defect‐free MOF‐801 membrane using a restricted‐coordination‐induced intergrowth strategy. The original MOF‐801 membrane showed a flux of approximately 7.8 L·m^−^^2^·h^−^^1^ and a rejection rate of 0.74% for MgCl_2_ under 5 bar pressure‐driven permeation test. After eliminating lattice defects, the optimized pore size increased the MgCl_2_ rejection rate to around 95%. When the pressure was further increased to 25 bar and then reduced back to 5 bar, the membrane structure remained stable, without deformation, and its performance showed no significant decline, further confirming the membrane's stability (Figure 10c). Compared to previously reported MOF membranes, this membrane exhibited enhanced flux due to its dense, defect‐free structure and thin membrane layer, resulting in a lower transport resistance. These properties significantly improved the flux, outperforming most advanced crystalline membranes, including zeolite and other MOF membranes (Figure 10d). The membrane's excellent desalination performance is a result of the synergistic effect of size sieving and dehydration energy barriers. The hydrated diameter of Mg^2^^+^ ions (8.56 Å) allow it to enter the 3.8 Å pore window of MOF‐801, indicating partial dehydration of the hydrated ion during entry into the MOF pores. Due to the electrostatic attraction between H_2_O and Mg^2^^+^, it is difficult for the ion to dehydrate during passage through the pores. In contrast to H_2_O, Mg^2^^+^ requires a higher activation energy to pass through the pores, contributing to the membrane's excellent desalination performance (Figure 10e).

MOF‐808 membranes have recently emerged as promising candidates for desalination applications and emerging contaminant removal. In their pioneering work, Liu et al. [83] fabricated the MOF‐808 membrane that showed only 6.3% NaCl rejection, yet exhibited excellent dye separation performance, rejecting over 99% of various organic dyes and maintaining stable performance over 96 h of continuous operation. Building on this, the same group later developed an in situ grown MOF‐808 membrane on a 100 nm α‐Al_2_O_3_ substrate for antibiotic/salt mixture separation [97]. This ultrathin membrane (0.89 µm) demonstrated a high‐water flux of ∼15 L·m^−^^2^·h^−^^1^, although its Na^+^ rejection remained low at 0.5%. Remarkably, when the membrane thickness was increased to 7.1 µm, the Na^+^ rejection rate increased to 69.7%, while the water flux decreased significantly to 0.21 L·m^−^^2^·h^−^^1^. This pronounced trade‐off between selectivity and flux with varying membrane thickness highlights the critical role of microstructural tuning for targeting both salts and new pollutants (e.g., antibiotics, dyes, and other emerging organic contaminants).

In addition to pure membranes, Zr‐MOF nanoparticles have been widely used as functional fillers in MMMs to enhance desalination performance. Table 3 summarizes the significant advancements in Zr‐MOF‐based MMMs for desalination. Zhu et al. [124] developed a Zr‐MOF nanofiltration MMM with a fish‐net‐like structure based on the MOF positioning strategy. Through a combined process of vacuum‐assisted filtration and interface polymerization, the researchers precisely anchored UiO‐66‐NH_2_ nanocrystals in the polyamide selective layer, successfully constructing a TFN composite membrane with high water permeability. Thanks to the interface voids formed by UiO‐66‐NH_2_ and the fish‐net‐like surface, the water flux of this membrane reached 123.2 L·m^−^^2^·h^−^^1^, which was 112.4% higher than that of the unmodified membrane, while maintaining excellent binary salt retention (Na_2_SO_4_ retention of 97.5%) and monovalent/divalent salt selectivity (NaCl/Na_2_SO_4_ selectivity coefficient of 31.9%). This high performance is attributed to the synergistic mechanism of MOF positioning, the expanded free volume of polyamide provides a rapid water transport channel, while the continuous strip structure connected by MOF nodes significantly increases the effective surface area. Similarly, Liu et al. [125] engineered a UiO‐66‐NH_2_‐based TFN nanofiltration membrane via a surface functionalization strategy employing stearoyl chloride. Owing to the supplementary nanochannels introduced by the UiO‐66‐NH_2_‐PC fillers and the optimized interfacial microenvironments within the polyamide matrix, the resultant membrane exhibited a pure water permeance of 49.6 L·m^−^^2^·h^−^^1^, marking a ∼53% augmentation relative to the unmodified thin‐film composite (TFC) control. Concurrently, the membrane sustained a robust MgSO_4_ rejection efficiency of approximately 90%. This performance enhancement is ascribed to the superior dispersibility of the nanoparticles in the organic phase; the long‐chain alkyl functionalization effectively mitigates nanoparticle agglomeration, thereby augmenting water permeability while preserving the membrane's separation selectivity. Bonnett et al. [126] fabricated nanocomposite RO membranes by incorporating PCN‐222 MOF nanorods modified with varying ratios of myristic acid (MA) into the aqueous phase monomer solution prior to interfacial polymerization. Results showed that TFN membranes containing PCN‐222 nanorods with a high MA loading (MA:TCPP ratio of 10:1) exhibited a water flux of 8.0 L·m^−^^2^·h^−^^1^ at a low loading of 0.01 wt.%, with NaCl rejection reaching 96.0%, approximately 95% higher than that of the pristine PA control membranes. This enhancement was attributed to the tunable pore size and the formation of rapid water transport pathways at the nanorod‐polymer interface, where the dense layer of myristic acid groups acted as gatekeepers to inhibit the transport of Na^+^ ions while facilitating water permeation.

**TABLE 3:** By introducing Zr‐MOF nanoparticles into the polymer matrix, the flux and selectivity were simultaneously improved.

Membrane structure	Water flux (L·m^−^^2^·h^−^^1^)	Salt rejection	Separation mechanism	Refs.
UiO‐66‐NH_2_/TFN (Organic phase loading)	87.86	Na^+^ rejection 99.09%, 500 h flux decreased<5%	Size sieving, electrostatic repulsion, monomer adsorption, reaction within MOFs pores	[73]
PA/UiO‐66/PES	1.36	NaCl rejection has been increased to 94.3%	The screening effect of nanochannels, the improvement of membrane hydrophilicity and the regulation of surface structure	[67]
MOF‐Glucose‐PDA/PSf	31.6	Na_2_SO_4_ rejection 99.8%	Size sieving, electrostatic repulsion and hydrophilic channels	[75]
UiO‐66‐F_4_/TFC	2.32	Na^+^ rejection 97.5%	By fluorinating nanochannels, water clusters are disrupted, rapid water molecule transport is promoted, and salt ions are retained simultaneously	[77]
PEI‐UiO‐66‐NH_2_/GO	2.37	Sr^2^^+^ rejection >90% (Multiple ion coexist), Cs^+^ rejection ≈40%, Sr^2^^+^/Cs^+^ Separation coefficient 12	Size screening (Sr^2^^+^ Hydration radius 0.50 nm > Cs^+^ 0.33 nm), PEI‐ZrO variety selective ligand	[132]
PCN‐224‐PA/TFN	20	Cl^−^/SO_4_^2^^−^ Separation coefficient 44 (Unmodified membrane 8.2)	Phytic acid pore expansion (0.6→0.9 nm) Negative surface reinforcement Donnan effect	[70]

**FIGURE 11:**
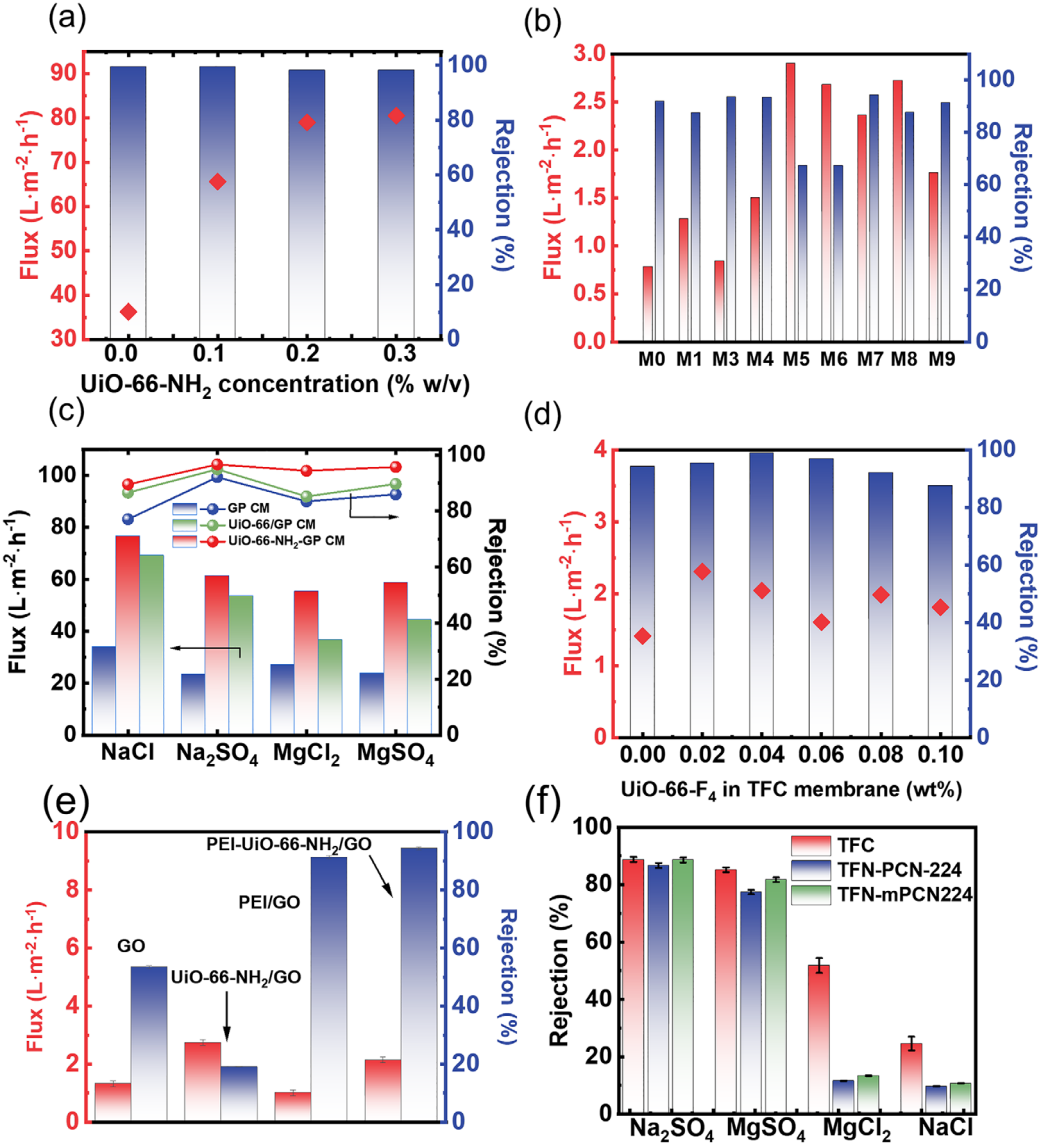
Desalination performance of Zr‐MOF membranes in pressure driven process. (a) NaCl separation performance of TF. N membranes with different UiO‐66‐NH_2_ concentrations in aqueous phase [73]. (b) The flux and rejection of PA/UiO‐66/PES composite membranes [67]. (c) Separation performance of different UiO‐66 composite membranes for saline solutions [75]. (d) Separation performance of UiO‐66‐F_4_/TFN membranes [77]. (e) Strontium separation performance of GO, UiO‐66‐NH_2_/GO, PEI/GO, and PEI‐UiO‐66‐NH_2_/GO membranes [78]. (f) The rejection of the TFC, TFN‐PCN‐224(1.5), and TFN‐mPCN‐224(1.5) membranes for different kinds of inorganic salt solutions [70].

Chen et al. [73] used IP to prepare four types of TFN membranes, systematically exploring the introduction strategies and loading amounts of Zr‐MOF nanoparticles (UiO‐66 and amino functionalized UiO‐66‐NH_2_) in aqueous and organic phases and their effects on membrane performance. Results showed that TFN membranes with UiO‐66‐NH_2_ in the organic phase exhibited a water flux of 87.86 L·m^−^^2^·h^−^^1^ at a 0.2% (w/v) concentration, with Na^+^ rejection up to 99.09%, approximately 120% higher than that of conventional polyamide TFC membranes (Figure 11a). During a 500‐h continuous operation with 3.5 wt.% NaCl, the flux decay rate was under 5%, demonstrating excellent anti‐fouling performance. This improvement was attributed to the significant slowing of the IP rate in the organic phase by the MOF particles, resulting in a thinner, more porous polyamide layer that reduced mass transfer resistance. Subsequently, Trinh et al. [67] successfully prepared a PA/UiO‐66/PES composite membrane by controlling the loading of UiO‐66 and the IP crosslinking process. Under 2 bar operating pressure, the optimized composite membrane exhibited an increase in NaCl rejection from 91.9% to 94.3%, and water flux from 1.24L·m^−^^2^·h^−^^1^ to 5.44 L·m^−^^2^·h^−^^1^ (Figure 11b). This demonstrates that the incorporation of UiO‐66 significantly enhanced the flux of traditional PA membranes while maintaining a high rejection rate. Liu et al. [75] employed a biomimetic strategy to design a 3D MOF‐Glucose‐PDA composite membrane, significantly enhancing both separation efficiency and anti‐fouling properties. The study innovatively used the synergistic effect of dopamine (PDA) and glucose to construct a dual‐functional layer for molecular sieving and fouling resistance on the surface of a PSF substrate. Under 5 bar operating pressure, this membrane exhibited 99.8% Na_2_SO_4_ rejection and water flux of 158 L·m^−^^2^·h^−^^1^, 108% improvement over traditional polyamide membranes (Figure 11c).

Sun et al. [77] significantly optimized the performance of Zr‐MOF‐based MMMs using a fluorination strategy. By introducing fluorinated UiO‐66 nanoparticles into the polyamide separation layer on a PVDF substrate via interfacial polymerization, they created a novel UiO‐66‐F4/TFN membrane. The optimized UiO‐66‐F4/TFN membrane achieved a water flux of 2.32 L·m^−^^2^·h^−^^1^, a 64% increase over the original TFC membrane, with Na^+^ rejection improved to 97.5% (Figure 11d). To address the selective separation of radioactive nuclides, Li et al. [78] developed a composite membrane based on UiO‐66‐NH_2_ and GO modified by polyethyleneimine (PEI). The membrane showed excellent selective rejection of Sr^2^^+^ in the presence of K^+^, Ca^2^^+^, and Mg^2^^+^, with Sr^2^^+^ rejection remaining above 90% while Cs^+^ rejection was only around 40%, achieving a Sr^2^^+^/Cs^+^ separation factor of 12. This high selectivity is attributed to the complexation of PEI's amine groups with the ‐Zr‐O clusters of UiO‐66‐NH_2_, selectively binding Sr^2^^+^. Additionally, the ordered channels of UiO‐66‐NH_2_ preferentially block Sr^2^^+^, which has a larger hydrated radius, while smaller hydrated Cs^+^ ions can pass through, governed by the size‐sieving mechanism (Figure 11e). Zhu et al. [70] developed a high‐performance Zr‐MOF‐based mixed‐matrix NF membrane using a phytate functionalization strategy. By combining EPD and VF‐IP, they successfully incorporated phytate‐modified PCN‐224 nanoparticles into a polysulfone‐based membrane, resulting in a PCN‐224‐PA/TFN composite membrane with functionalized nanopores. The resulting membrane achieved water flux of 20 L·m^−^^2^·h^−^^1^, 56.4% improvement over the unmodified membrane, and exhibited excellent anion selectivity, with a Cl^−^/SO_4_^2^^−^ separation factor of 44 (compared to 8.2 for the unmodified membrane). This high performance is attributed to the synergistic mechanism of phytate functionalization, where enlarged MOF pores preferentially allow rapid transport of Cl^−^, while the highly negative surface efficiently rejects SO_4_^2^^−^ through the Donnan effect (Figure 11f).

### 4.2 Forward Osmosis Desalination

**FIGURE 12:**
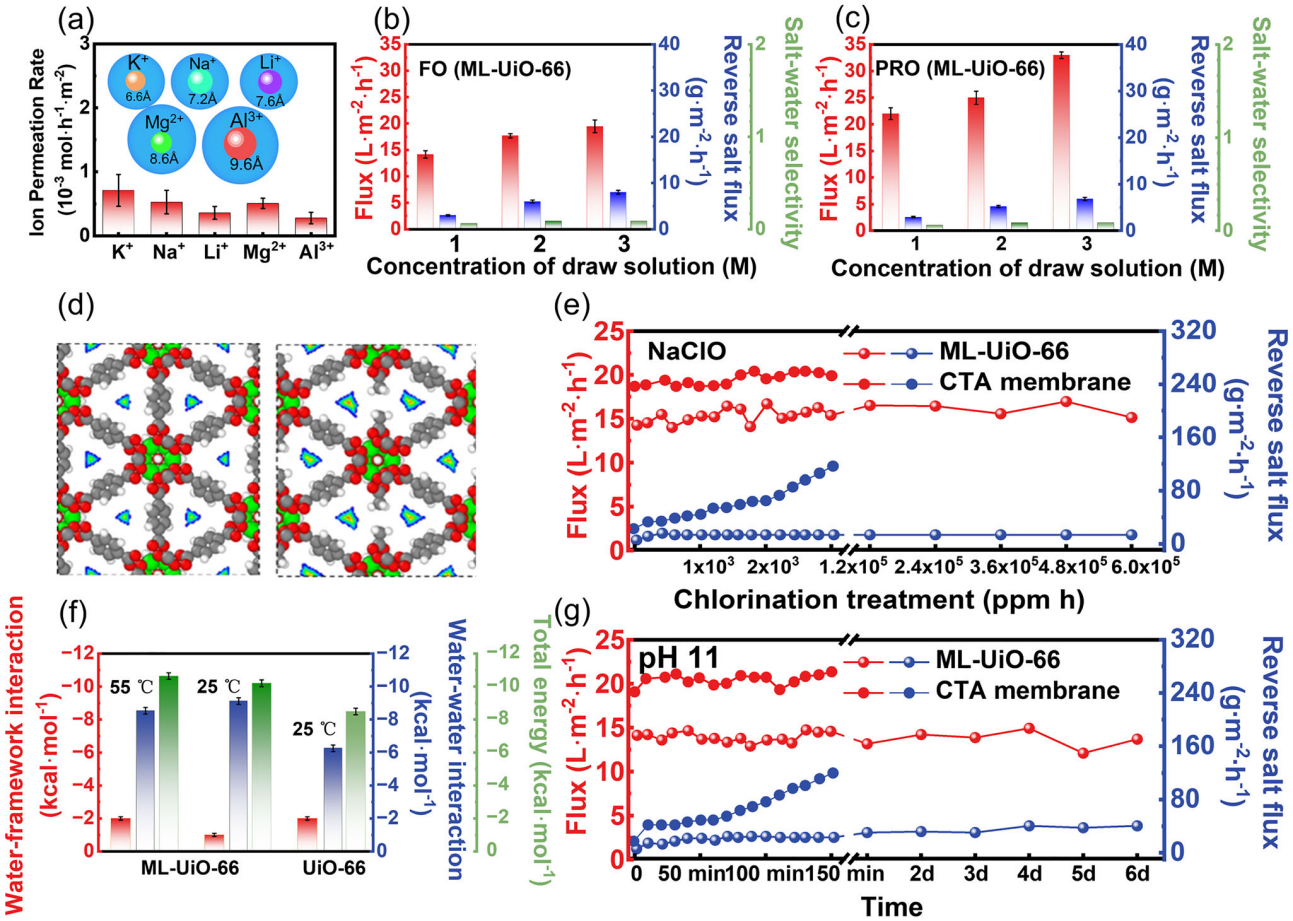
Desalination performance of Zr‐MOF membranes in FO process. (a) Ion permeation rate of K^+^, Na^+^, Li^+^, Mg^2+^, and Al^3+^ through the UiO‐66 membrane grown via microwave synthesis [85]. (b) Water flux, reverse salt flux, and salt‐water selectivity as a function of draw solution concentration via FO mode for the ML‐UiO‐66 membranes [92]. (c) Water flux, reverse salt flux, and salt‐water selectivity as a function of draw solution concentration via PRO mode for the ML‐UiO‐66 membranes [92]. (d) Cross‐sectional schematic diagram of nanochannels for water molecule [92] (white: hydrogen, grey: carbon, green: zirconium, red: oxygen). (e) Total interaction energy, water (water interaction energy, and water (framework interaction energy of UiO‐66 (25°C) and ML‐UiO‐66 (25°C, 55°C) membranes [92]. (f) Water flux and reverse salt flux under strongly alkaline operating conditions [92]CTA: Cellulose triacetate. (g) Water flux and reverse salt flux under high NaClO operating conditions [92].

FO membrane separation utilizes the osmotic pressure difference as the driving force, allowing water molecules to pass from the low osmotic pressure side to the high osmotic pressure side through a selective membrane. Due to its theoretically lower energy consumption, FO is considered a promising and energy‐efficient desalination method [50]. In recent years, pure Zr‐MOF membranes have attracted considerable research attention for FO desalination applications. Wei et al. [85] used microwave‐assisted synthesis to rapidly fabricate UiO‐66 crystal membranes on porous supports. When evaluated in FO mode, the membrane achieved a water flux of up to 0.16 L·m^−^^2^·h^−^^1^, with a Na^+^ rejection rate consistently maintaining 99.6% during a 700‐h long‐term stability test (Figure 12a). Recently, Dong et al. [92] developed a ceramic‐based UiO‐66 nanopore membrane with intraframe defects (i.e., missing linkers, ML‐UiO‐66) for FO desalination. The water flux of the ML‐UiO‐66 membrane was 14.3 ± 0.6 L·m^−^^2^·h^−^^1^, which is higher than that of the UiO‐66 membrane (9.9 ± 1.2 L·m^−^^2^·h^−^^1^). In FO mode using 1 M NaCl as the draw solution, the water flux of the ML‐UiO‐66 membrane was 44%–56% higher than that of the UiO‐66 membrane, and in pressure‐retarded osmosis (PRO) mode, the water flux increased by 101%–114% (Figure 12b,c). The reverse salt flux of the ML‐UiO‐66 membrane was very low (∼1.3 g·m^−^^2^·h^−^^1^), much lower than most commercial polymer membranes. In FO mode, the salt rejection of the ML‐UiO‐66 membrane was as low as ∼0.027, significantly outperforming other membrane materials. Under high‐temperature conditions (25‐55°C), the water flux of the ML‐UiO‐66 membrane significantly increased, from 14.3 L·m^−^^2^·h^−^^1^ to 49.7 L·m^−^^2^·h^−^^1^, with only a slight increase in reverse salt flux, indicating better thermal stability and chemical resistance. These results are attributed to the introduction of linker‐missing defects, which increased the pore size of the MOF nanochannels from 3.86 Å to 4.40 Å (Figure 12d). The enlarged pore size of the MOF nanochannels also lowered the energy barrier for water transport, accelerating water flux (Figure 12e). The ML‐UiO‐66 membrane demonstrated significantly better stability under extreme conditions, such as strong alkali and high chlorine concentrations, outperforming commercial polymer membranes. Additionally, it showed good operational stability and structural integrity when treating real industrial petrochemical wastewater (Figure 12f,g).

Zr‐MOF‐based MMMs for FO desalination have also been widely studied. In 2017, Han et al. [123] prepared a TFN membrane incorporating UiO‐66. In the PRO mode, the water flux of the TFN membrane was 40% higher than that of the TFC membrane, reaching 36.7 L·m^−^^2^·h^−^^1^. When the draw solution concentration was increased to 2 M NaCl, the water flux further increased to 51.3 L·m^−^^2^·h^−^^1^. In the FO mode, the water flux was 25% higher than that of the TFC membrane, achieving 20.7 L·m^−^^2^·h^−^^1^, upon increasing the NaCl concentration to 2 M, the water flux reached 27 L·m^−^^2^·h^−^^1^. The TFN membrane exhibited a salt rejection of approximately 95%, and the high‐water stability of UiO‐66 ensured both structural integrity and long‐term performance stability.

**FIGURE 13:**
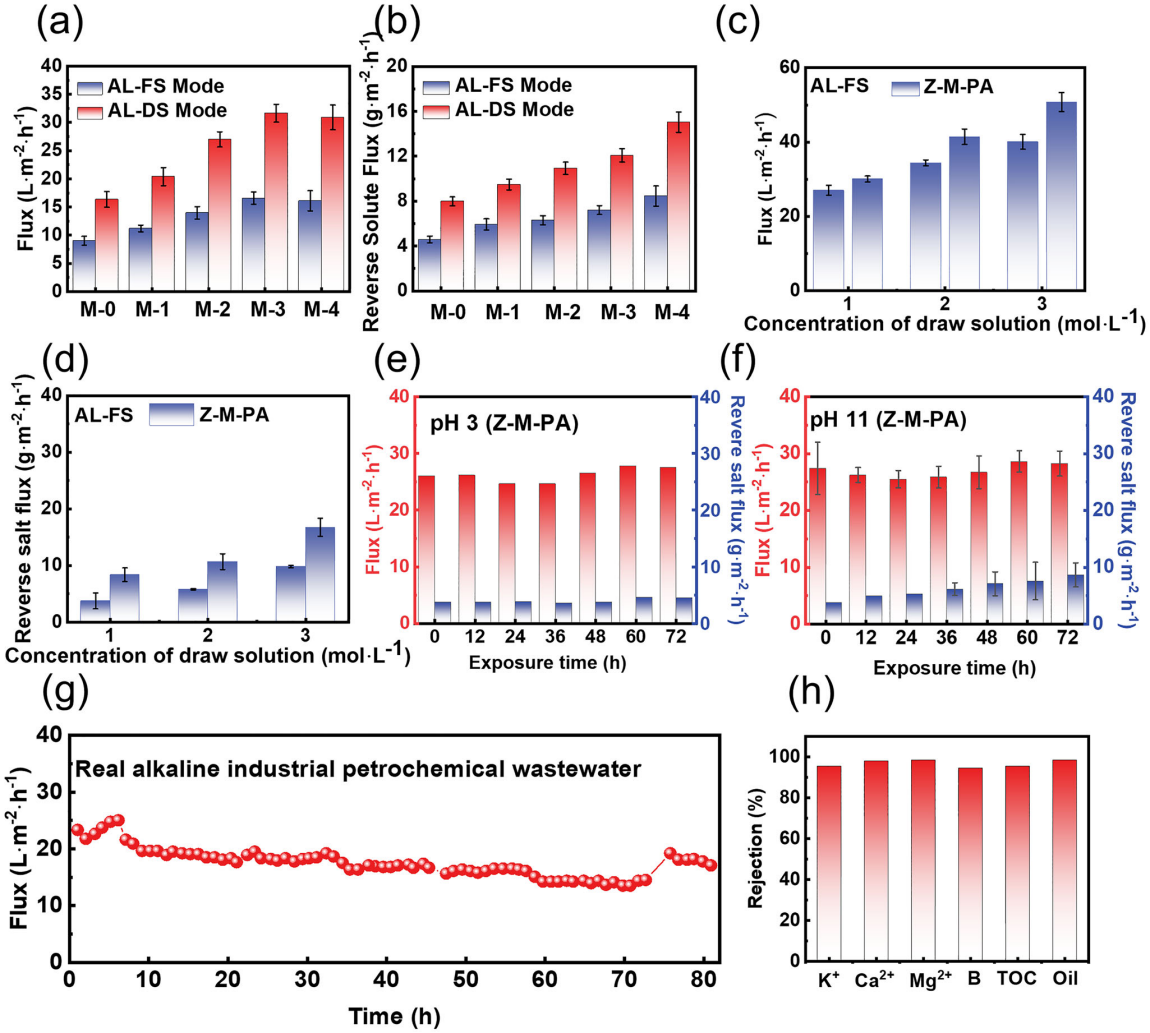
Desalination performance of Zr‐MOF membranes in FO process. (a) Water flux and (b) Reverse solute flux of the TFC membranes in long‐term FO chemical stability test under both AL‐FS and AL‐DS modes; AL‐FS: Active Layer facing Feed Solution, AL‐DS: Active Layer facing Draw Solution [68]. (c,d) Water treatment performance of TFN membrane with UiO‐66‐NH_2_ interlayer [36]. (e,f) Performance of TFN membrane with Zr‐MOF interlayer under harsh conditions; Z‐M‐PA: Zr‐based ceramic substrate with MOF interlayer and Polyamide selective layer [36]. (g,h) Operation performance (water flux) and stability for the treatment of real industrial petrochemical wastewater using TFN membrane with Zr‐MOF interlayer; TOC: Rejections of various components [36].

Xie et al. [68] used IP to fabricate a dual‐functional polyamide‐TFC (PA‐TFC) membrane modified with a Ti‐modified MOF (Ti‐UiO‐66). The water flux of this membrane in both FO and PRO modes was significantly higher than that of the unmodified PSF support membrane. In FO mode, the water flux increased from 8.99 L·m^−^^2^·h^−^^1^ for the base membrane to 16.71 L·m^−^^2^·h^−^^1^, an 85.9% improvement. In PRO mode, the water flux increased from 16.38 L·m^−^^2^·h^−^^1^ to 31.57 L·m^−^^2^·h^−^^1^, a 92.7% enhancement (Figure 13a). These results demonstrate that the membrane effectively reduces internal concentration polarization (ICP) during FO operation. Furthermore, the reverse solute flux of the Ti‐UiO‐66‐modified membrane was significantly lower than that of the UiO‐66 membrane (Figure 13b). More recently, Dong et al. [36] developed a composite membrane by constructing a PA separation layer on a support that had been pre‐grown with a UiO‐66‐NH_2_ intermediate layer via interfacial polymerization. This membrane exhibited excellent water flux (27.38 L·m^−^^2^·h^−^^1^) and very low reverse salt flux (3.45 g·m^−^^2^·h^−^^1^) (Figure 13c,d). The ceramic‐based FO membrane with the Zr‐MOF intermediate layer not only outperformed most PA membranes doped with MOFs in terms of water and salt transport properties, but also exhibited significant potential for practical applications in harsh environments, such as acidic (pH = 3) and alkaline (pH = 11) solutions (Figure 13e,f). Additionally, when used in the FO process to treat real alkaline industrial wastewater (pH = 8.6), the membrane maintained a stable water flux of 22.62 L·m^−^^2^·h^−^^1^ during the first 10 h. From 10 to 60 h, the water flux decreased from 20.18 L·m^−^^2^·h^−^^1^ to 14.35 L·m^−^^2^·h^−^^1^, remaining relatively stable over the next 12 h (Figure 13g). During this period, the membrane also demonstrated high rejection rates for various contaminants, including K^+^ (96.12%), Ca^2^^+^ (99.12%), Mg^2^^+^ (99.66%), B (94.93%), Total Organic Carbon (96.73%), and oil (100%) (Figure 13h).

### 4.3 Pervaporation Desalination

In PV desalination, water molecules selectively permeate through a dense membrane layer, evaporate on the permeate side, and condense, achieving near‐perfect salt rejection. However, many high‐performance PV membranes (e.g., zeolite membranes) are still limited by the trade‐off between water flux and selectivity at the micron scale. In contrast, Zr‐MOFs have gained significant attention in the field of PV desalination due to their precisely tunable sub‐nanometer pore sizes, rich channel structures, and unique chemical environments derived from metal clusters and organic linkers [63].

**FIGURE 14:**
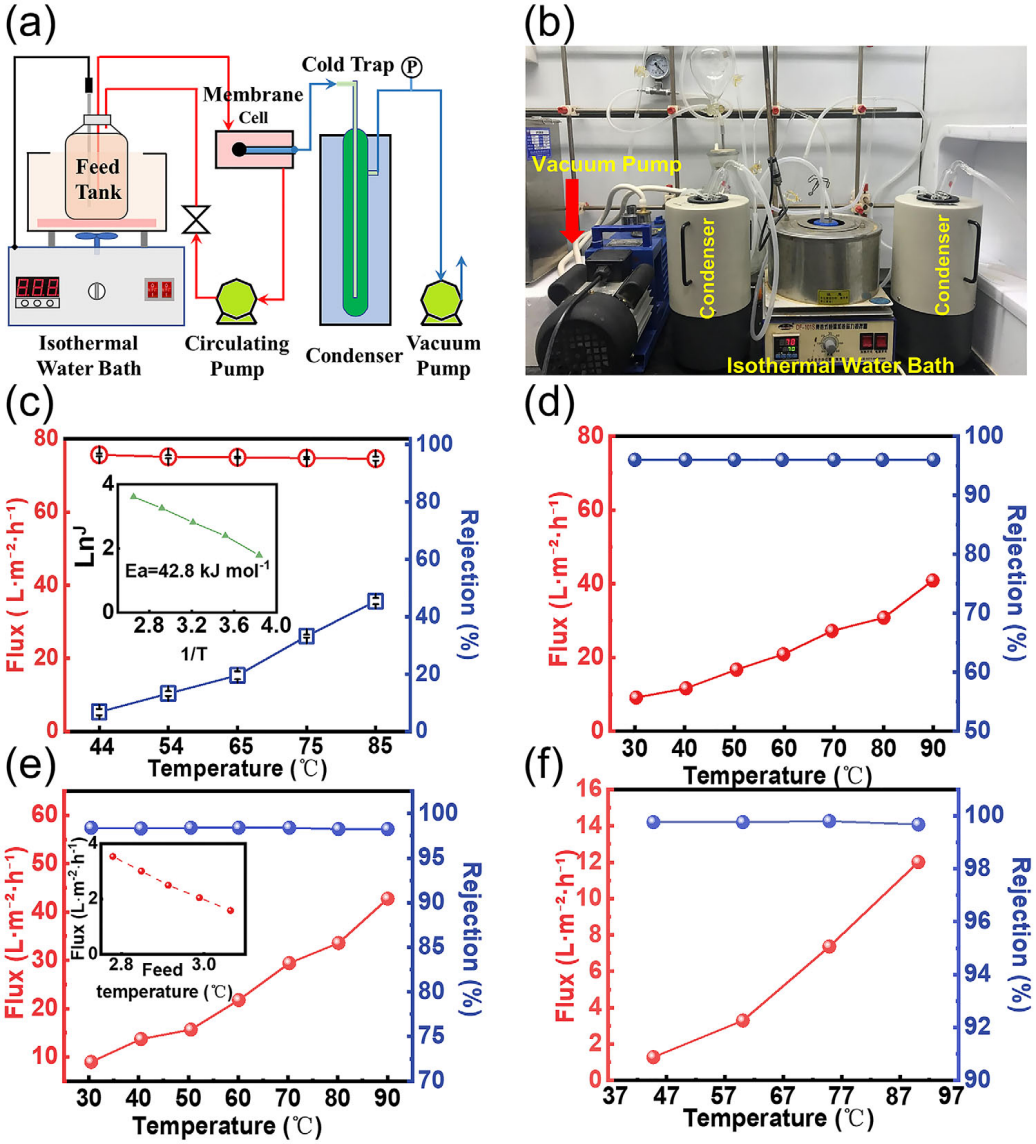
Desalination performance of Zr‐MOF membranes in PV process. (a) Schematic diagram of the pervaporation setup, (b) photograph [88]. (c) Water flux and salt rejection of UiO‐66 membrane under various feed temperatures [88]. (d) Water flux and salt rejection under different feed temperatures of ceramic‐based MOF membranes [91]. (e) Water flux and salt rejection as a function of feed temperature during the PV process of robust ultrathin UiO‐66/ML‐UiO‐66 membranes [133]. (f) Water flux and ion rejection of the UiO‐66‐NH_2_ membrane prepared on APTES modified α‐Al_2_O_3_ tube for seawater desalination as a function of the feed concentration at 348 K [87].

**FIGURE 15:**
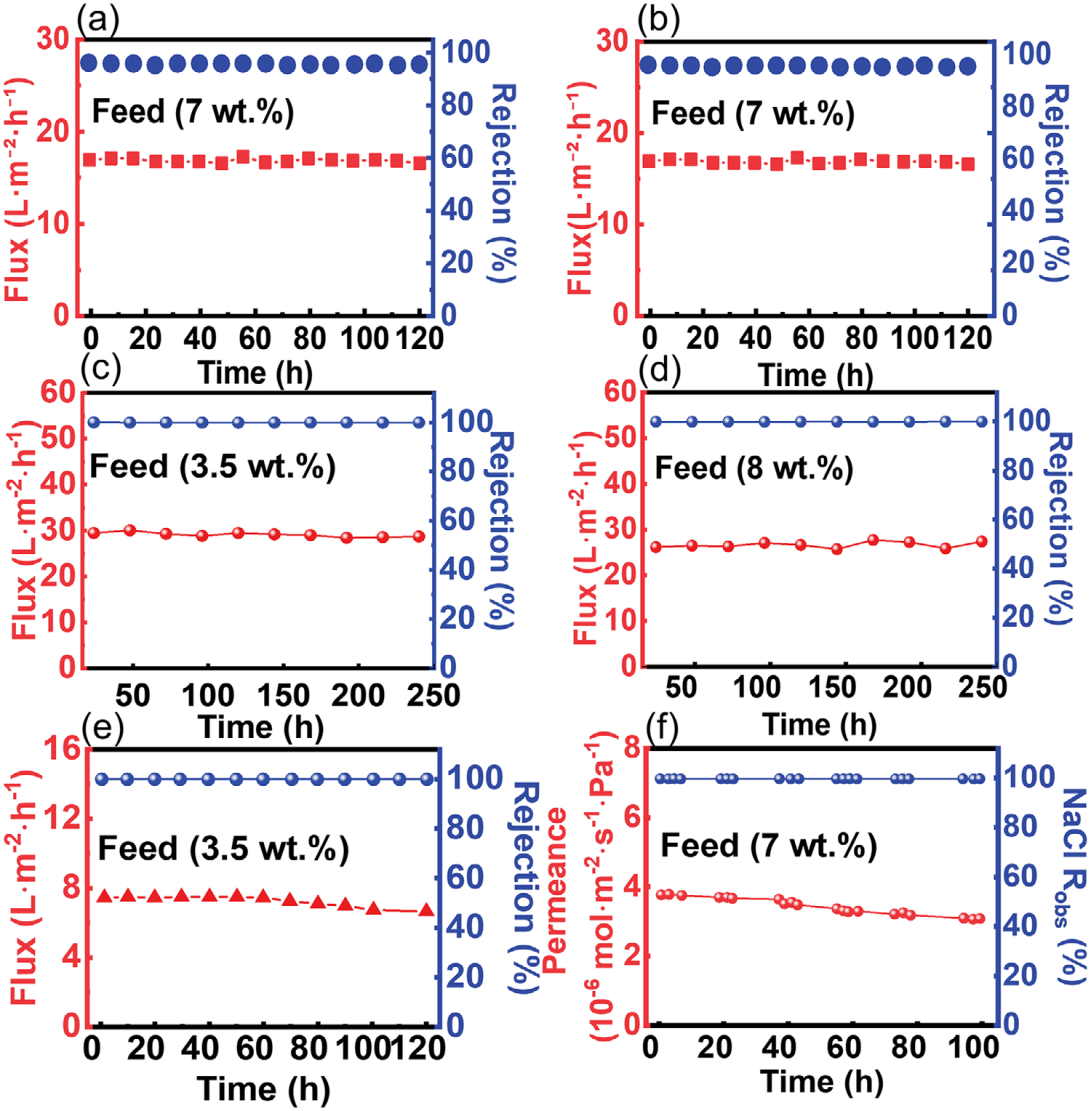
Desalination performance of Zr‐MOF membranes in PV process. (a) Hypersaline water treatment operation stability of the UiO‐66 membrane [88]. (b) After soaking at acidic solution desalination performance comparison of UiO‐66 membrane [91]. (c,d) Long‐term operating desalination performance of robust ultrathin UiO‐66/ML‐UiO‐66 membranes [133]. (e) Water flux and ion rejection of the UiO‐66‐NH_2_ membrane prepared on APTES modified α‐Al_2_O_3_ tube for desalination of 3.5 wt.% seawater for 120 h at 348 K by PV [87]. (f) Continuous PV test of the UIO‐66‐NH_2_/BTESE membrane for desalination of 7 wt.% NaCl solutions at 60°C [130].

Dong's group conducted a systematic study on the use of Zr‐MOFs pure membranes for PV desalination (Figure 14a,b). Dong et al. [88] were the first to apply UiO‐66 membranes, synthesized on a porous support via in situ solvothermal growth, for PV desalination. At a feed temperature of 65°C, the membrane achieved a water flux of 37.4 L·m^−^^2^·h^−^^1^ and exhibited near‐perfect Na^+^ rejection, with a rejection rate of 99.9% (Figure 14c). The membrane demonstrated excellent long‐term operational stability and anti‐fouling performance under high temperature, high salt concentration, and acidic/alkaline conditions (Figure 15a). Subsequently, Dong et al. [91] explored the applicability of Zr‐MOF membranes in PV desalination using real industrial wastewater. During PV tests with a 3.5 wt.% NaCl solution, the water flux of the UiO‐66 membrane increased markedly from 9.23 to 41.35 L·m^−^^2^·h^−^^1^ as the temperature was raised from 30°C to 90°C, while Na^+^ rejection remained consistently high at 99.9% (Figure 14d). The membrane also demonstrated exceptional ion selectivity, achieving rejection rates of 97.62% for K^+^, 99.71% for Na^+^, 99.45% for Ca^2^^+^, and 99.94% for Mg^2^^+^. Notably, the membrane showed no significant decrease in desalination efficiency under extreme pH values, high oxidative conditions, or ultrasonic treatments, demonstrating its strong adaptability and application potential under harsh conditions. Moreover, the UiO‐66 membranes displayed excellent stability over 10 days of operation, with consistently stable water flux (25.83∼27.31 L·m^−^^2^·h^−^^1^) and salt rejection (∼99.9%), as well as an intact membrane structure during the PV process (Figure 15b).

To further enhance the separation performance, Dong et al. [133] combined molecular‐level crystal defect engineering with ultrathin membrane fabrication, developing a novel ML‐UiO‐66 membrane. Under 70°C, during continuous testing with 35 g·L^−^^1^ and 80 g·L^−^^1^ NaCl solutions for 10 days, the membrane's water flux remained stable between 20.8 and 29.8 L·m^−^^2^·h^−^^1^, with a constant Na^+^ rejection rate of 99.9% (Figure 15c,d). When the separation temperature was increased to 90°C, the water flux reached a maximum of 45.1 ± 1.6 L·m^−^^2^·h^−^^1^, with the rejection rate remaining unaffected (Figure 14e). Huang et al. [87] prepared UiO‐66‐NH_2_ membranes that exhibited excellent performance during PV desalination. As the feed temperature increased from 318 to 363 K, the water flux increased from 1.5 to 12.1 kg·m^−^^2^·h^−^^1^ due to the enhanced water permeation driving force, while exhibiting high rejection rates (>99.84%, some reaching 100%) for various ions (Na^+^, NH_4_^+^, K^+^, Mg^2^^+^, Ca^2^^+^, Cl^−^, NO_3_^−^, F^−^) (Figure 14f). Further stability tests of the UiO‐66‐NH_2_ membrane under 348 K and 3.5 wt.% seawater conditions showed that after 120 h, both water flux (7.5 kg·m^−^^2^·h^−^^1^) and ion rejection (∼99.7%) remained stable, indicating the membrane's potential for long‐term efficient desalination (Figure 15e). XRD analysis further confirmed that the membrane's crystalline structure remained unchanged after 200 h of use, demonstrating its excellent stability.

Zr‐MOFs have also been used as functional fillers in MMMs for PV desalination. Liu et al. [130] developed a novel UiO‐66‐NH_2_ /BTESE MMM for PV desalination. Continuous PV desalination tests with a 7 wt.% NaCl solution at 60°C showed that during a 100‐h continuous operation, the membrane's NaCl rejection remained above 99.9% without significant change (Figure 15f). Although membrane fouling and concentration polarization led to a 16% decrease in water flux after 100 h, the flux remained above 3.1 × 10^−^^6^ mol·m^−^^2^·s^−^^1^·Pa^−^^1^, further proving the membrane's superior long‐term stability. Recently, Dong et al. [113] fabricated MOF membranes via nanocrystal‐induced secondary growth combined with surfactant post‐treatment, enabling precise regulation of ligand vacancies and reducing the water transport activation energy to 3.69 kJ·mol^−^^1^. The membrane achieved >99.9% salt rejection with a water flux of 35.6 L·m^−^^2^·h^−^^1^ and maintained stable performance under harsh conditions.

### 4.4 Other Desalination Processes With Zr‐MOF Membranes

Beyond pressure‐driven membrane separation, FO and PV desalination processes, researchers have also proposed innovative desalination strategies based on Zr‐MOF membranes, including MD, and diffusion dialysis. Cao et al. [76] used vacuum‐assisted filtration to embed UiO‐66‐NH_2_ and silver nanowires into a GO composite membrane, creating a novel Janus‐modified composite membrane for MD desalination. Under intermittent voltage application (2 V), the membrane's permeation conductivity remained stable at 1.9 µS∙cm^−1^ over 48 h, with a stable water flux of around 19.6 L·m^−^^2^·h^−^^1^. In a long‐term 170 h stability test, the membrane maintained a permeation conductivity of less than 20 µS∙cm^−1^, demonstrating its excellent stability and anti‐wetting capability. Jin et al. [129] fabricated a novel MMM for DCMD by depositing a MOF‐801@GO composite onto a PVDF substrate via vacuum‐assisted filtration. The additional nanochannels created by MOF‐801 nanoparticles within the GO interlayers significantly enhanced the vapor transport rate. At a feed temperature of 60°C, the membrane achieved a water flux of 22.94 L·m^−^^2^·h^−^^1^ with a salt rejection rate of 99.99%. When the temperature was increased to 70°C, the flux further increased to 29.24 kg·m^−^^2^·h^−^^1^ while maintaining a salt rejection above 99.99%. During a long‐term 72 h test, both the water flux and salt rejection showed no significant decline.

Kalla et al. [65] synthesized PVDF‐modified HF membranes using phase inversion technology. Under the optimal experimental conditions (65°C, 1 LPM feed flow rate, and 1.5 wt.% NaCl), the membrane achieved a maximum water flux of 26.78 L·m^−^^2^·h^−^^1^. In DCMD, the membrane exhibited salt rejection rates greater than 99.8% for KCl, MgSO_4_, and CaSO_4_. Under optimized operating conditions, the membrane maintained high water flux and salt rejection (>99.9%) during 80 h of long‐term operation. Li et al. [74] used casting to fabricate a UiO‐66‐NH_2_ and PDMS hybrid separation membrane. After soaking the membrane in 1 m salt solution for 240 h, XRD results showed that the membrane structure remained unchanged. When the soaking time was extended to 10 days, the separation performance did not degrade. The addition of UiO‐66‐NH_2_ significantly enhanced the Na^+^/Ca^2^^+^ selectivity, with the separation factor increasing from 4.03 for the original PDMS membrane to 11.2, a 178% improvement. Jin et al. [121] further evaluated the desalination performance of the pure UiO‐66‐NH_2_ membrane, prepared via epitaxial growth, using a diffusion dialysis method. The membrane exhibited excellent mono‐/divalent ion selectivity. In the mixed salt system, the permeation rate of K^+^ was 0.52 mol·m^−^^2^·h^−^^1^, with a K^+^/Mg^2^^+^ selectivity as high as 98; the Na^+^/Mg^2^^+^ selectivity reached 76.9; the permeation rate of Li^+^ was approximately 0.38 mol·m^−^^2^·h^−^^1^, corresponding to a Li^+^/Mg^2^^+^ selectivity of 63.9. During a continuous 100‐h operation, the separation performance for K^+^/Mg^2^^+^ and Li^+^/Mg^2^^+^ remained stable (K^+^ selectivity ∼76, Li^+^ selectivity ∼56), and no noticeable changes in membrane structure or morphology were observed.

Zr‐MOF membranes have garnered extensive attention in water desalination, with performance testing under practical scenarios, high salinity, extreme pH, and complex matrices, demonstrating their technical feasibility. These membranes utilize their precisely tunable pore sizes to directly perform physical sieving based on the size differences of hydrated ions or molecules, allowing small molecules (such as water) to pass through while retaining larger solutes (such as salt ions and dye molecules). Furthermore, Zr‐MOF membranes with specific surface charges can efficiently retain or selectively permeate ions with opposite charges through the Donnan effect, which involves electrostatic repulsion or attraction. This is crucial for achieving the selective separation of anions (e.g., Cl^−^/SO_4_^2^^−^) or cations (e.g., Na^+^/Ca^2^^+^).

## 5 Conclusion and Perspective

**FIGURE 16:**
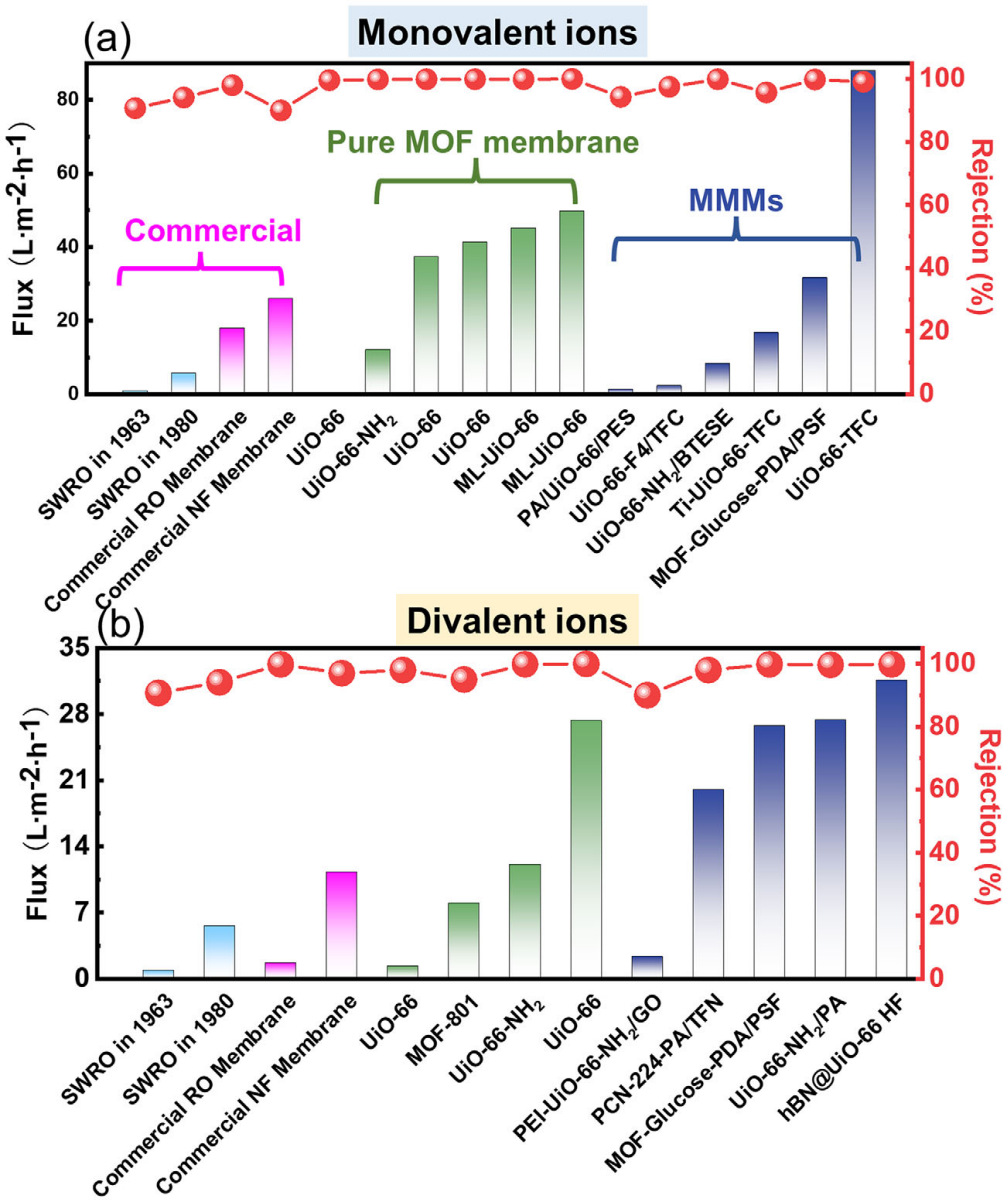
Performance comparison of state‐of‐the‐art Zr‐based MOF desalination membranes in terms of water flux and salt rejection for (a) monovalent and (b) divalent ions. Note, Commercial RO membrane [134], Commercial NF membrane [134]. Note: Divalent ions: Commercial RO membrane [135], 11. Commercial NF membrane [135], Saline water reverse osmosis (SWRO).

This review has provided a critical overview of recent advances in Zr‐MOF membranes for high‐efficiency desalination, with emphasis on the interaction between material chemistry, membrane fabrication strategies, and separation performance. We systematically examined representative Zr‐MOF platforms, UiO‐66 and its derivatives, MOF‐801, MOF‐808, and PCN‐224—highlighting how their pore architectures, defect chemistry, and surface functionalities dictate water transport and ion selectivity. We further categorized fabrication strategies for both pure crystalline membranes and Zr‐MOF‐based mixed‐matrix membranes, analyzing how synthesis routes influence membrane microstructure, defect distribution, and ultimately desalination performance across pressure‐driven processes, forward osmosis, and pervaporation. Collectively, these advances have promoted Zr‐MOF membranes to a performance level that approaches or even surpasses that of conventional polymeric desalination membranes. Many reported systems now achieve near‐complete rejection of monovalent salts (∼99.9%) while maintaining high water flux, effectively addressing the permeability–selectivity trade‐off that has historically constrained polymer‐based membranes (Figure 16). The milestone timeline presented in this review further illustrates the rapid evolution of Zr‐MOF desalination membranes from early proof‐of‐concept crystalline layers to state‐of‐the‐art ultrathin, defect‐engineered, and hybrid membrane structures capable of operating under harsh chemical and thermal conditions. Despite these encouraging achievements, the field remains at an early developmental stage, with several key challenges and opportunities demanding attention.

Current research efforts remain concentrated on UiO‐66‐type frameworks, underscoring both their versatility and the untapped potential of alternative Zr‐MOF structures. Expanding the material library beyond well‐established platforms, through linker chemistry diversification, pore topology engineering, and multimetallic cluster incorporation, will be essential to unlock new separation mechanisms and address emerging application needs, including the removal of new organic contaminants coexisting with salts in industrial wastewater. Developing low‐energy, green, and scalable synthesis routes for producing defect‐controlled Zr‐MOF membranes remains a central challenge [136]. Reducing solvent consumption, reaction temperature, and synthesis time, while maintaining membrane uniformity and crystallinity, will be important for industrial viability. Advanced defect engineering strategies, combined with support surface modification and controlled nucleation, will play a decisive role in constructing ultrathin, defect‐free membranes [137]. A deeper mechanistic understanding of how pore geometry, surface chemistry, and defect distribution govern ion dehydration, electrostatic exclusion, and water transport is still required. Bridging experimental observations with molecular simulations will enable rational, mechanism‐informed membrane design. Future studies should increasingly focus on membrane performance in real‐world scenarios, including seawater, high‐salinity industrial brines, and wastewater containing both salts and organic pollutants, where high salinity, extreme pH, oxidants, and complex matrices coexist. Long‐term stability, antifouling performance, and process integration studies will be indispensable for assessing practical feasibility. Ligand functionalization (e.g., amino, sulfonic acid, and phosphonate groups), bimetallic cluster incorporation, and hybrid MOF–polymer or MOF–COF architectures offer powerful routes to tailor selectivity, antifouling behavior, and chemical robustness. Particularly promising is the development of Zr‐MOF membranes capable of simultaneous salt removal and organic contaminant rejection, a critical requirement for treating industrial effluents containing dyes, pharmaceuticals, or petroleum hydrocarbons alongside high salt concentrations. The convergence of Zr‐MOF membrane science with artificial intelligence and machine learning holds great promise for accelerating materials discovery and membrane optimization [138]. Data‐assisted screening of MOF structures, predictive modeling of transport behavior, and intelligent process control could collectively drive the development of next‐generation and sustainable desalination membranes. In summary, Zr‐MOF membranes have demonstrated clear potential for desalination applications, offering a combination of stability, tunability, and separation performance unattainable with conventional materials. Continued progress at the interface of materials chemistry, membrane engineering, and process integration, coupled with a sustained focus on real‐world application challenges, will be crucial to fully realize their promise and enable their translation from laboratory‐scale innovation to practical, large‐scale water purification technologies.

## Conflicts of Interest

The authors declare no conflicts of interest.

## Data Availability

The data that support the findings of this study are available from the corresponding author upon reasonable request.
